# Characterization of the *Staphylococcus xylosus* methylome reveals a new variant of type I restriction modification system in staphylococci

**DOI:** 10.3389/fmicb.2023.946189

**Published:** 2023-03-08

**Authors:** Carolin J. Schiffer, Christian Grätz, Michael W. Pfaffl, Rudi F. Vogel, Matthias A. Ehrmann

**Affiliations:** ^1^Chair of Technical Microbiology, TUM School of Life Sciences, Technical University of Munich, Freising, Germany; ^2^Chair of Animal Physiology and Immunology, TUM School of Life Sciences, Technical University of Munich, Freising, Germany

**Keywords:** *Staphylococcus xylosus*, restriction modification systems, methylome, epigenetics, DNA methylation, methyltransferases (MTases)

## Abstract

Restriction modification (RM) systems are known to provide a strong barrier to the exchange of DNA between and within bacterial species. Likewise, DNA methylation is known to have an important function in bacterial epigenetics regulating essential pathways such as DNA replication and the phase variable expression of prokaryotic phenotypes. To date, research on staphylococcal DNA methylation focused mainly on the two species *Staphylococcus aureus* and *S. epidermidis*. Less is known about other members of the genus such as *S. xylosus*, a coagulase-negative commensal of mammalian skin. The species is commonly used as starter organism in food fermentations but is also increasingly considered to have an as yet elusive function in bovine mastitis infections. We analyzed the methylomes of 14 *S. xylosus* strains using single-molecular, real-time (SMRT) sequencing. Subsequent *in silico* sequence analysis allowed identification of the RM systems and assignment of the respective enzymes to the discovered modification patterns. Hereby the presence of type I, II, III and IV RM systems in varying numbers and combinations among the different strains was revealed, clearly distinguishing the species from what is known for other members of the genus so far. In addition, the study characterizes a newly discovered type I RM system, encoded by *S. xylosus* but also by a variety of other staphylococcal species, with a hitherto unknown gene arrangement that involves two specificity units instead of one (*hsdRSMS*). Expression of different versions of the operon in *E. coli* showed proper base modification only when genes encoding both *hsdS* subunits were present. This study provides new insights into the general understanding of the versatility and function of RM systems as well as the distribution and variations in the genus *Staphylococcus*.

## 1. Introduction

*Staphylococcus xylosus* is a Gram-positive, coagulase-negative commensal of mammalian skin with high biotechnological value, as it is commonly used as starter organisms in food fermentations (Ravyts et al., 2012; Leroy et al., 2017). However, over the past years, studies have associated *S. xylosus* with infections, i.e., bovine mastitis infections, as well (Supré et al., 2011; Condas et al., 2017). In addition, a growing number of studies is addressing the potential of coagulase-negative staphylococci (CoNS) to serve as reservoir for virulence-associated factors (Otto, 2013; Rossi et al., 2017; Heilmann et al., 2019; Marincola et al., 2021). Considering that spread of virulence and acquisition of antibiotic resistance genes are emerging topics nowadays (Karkman et al., 2018; Heilmann et al., 2019; Lindsay, 2019), it is of great concern that such genes can fuel pathogen evolution of highly pathogenic species such as *S. aureus* when transferred via horizontal gene transfer (HGT). Therefore, it is important to understand the probability and extent to which HGT occurs as well as the natural barriers that bacteria possess against HGT. One way, in which bacteria protect themselves from the uptake of exogenous, foreign DNA is by restriction modification (RM) systems. Active RM systems have been shown to be one of the major factors preventing inter- and intraspecies HGT (Tock and Dryden, 2005; Lindsay, 2014; Atack et al., 2018). Thereby, they shape bacterial genome evolution and impact host adaption mechanisms (Kobayashi, 2001; Park et al., 2022). The basic principle of distinguishing between foreign and own DNA is the site-specific modification of the individual DNA by methyltransferases combined with the expression of effective restriction endonucleases that recognize and cleave any unmodified, foreign DNA (Wilson and Murray, 1991; Sadykov, 2016).

Four major types of bacterial restriction (modification) systems (type I - IV) have been described to date. They are distinguished based on their enzymatic subunits, mechanism of action, DNA specificity/sequence recognition motifs as well as co-factor requirements and reaction conditions (Wilson and Murray, 1991; Loenen et al., 2014; De Ste Croix et al., 2017; Oliveira and Fang, 2021).

Type I systems are heterooligomeric complexes composed of three subunits, a methyltransferase (*hsdM*), modifying the host DNA by adding a methyl group in a specific sequence, a restriction endonuclease cleaving non-modified DNA (*hsdR*) and a specificity unit (*hsdS*) determining the recognition sequence of the system (Murray, 2000; Loenen et al., 2014; Gao et al., 2020). Hereby, *hsdM* and *hsdS* are usually transcribed from a common promoter, while *hsdR* is under the control of its own promoter (Murray, 2000). HsdS-HsdM complexes are active in methylation within the recognition sequence while HsdS-HsdM-HsdR complexes are active in restriction at unspecified sites on the unmodified DNA (Gao et al., 2020). Their cleavage sites are generally far from the methylation sites. How these sites are determined has not yet been fully clarified (Ishikawa et al., 2009).

Currently, type I RM systems are subdivided into five families (IA – IE) based on sequence homology and genetic complementation (Titheradge et al., 2001; Cooper et al., 2017). While *hsdM* and *hsdR* are very conserved within one family, with sequence similarity values reported between 70 - 90%, *hsdS* consists of two highly variable regions (Murray, 2000; Chin et al., 2004; Monk et al., 2015; Cooper et al., 2017). These variable regions encode the target recognition domains (TRDs) of HsdS, each of them specifying one half of the bipartite recognition motif (Murray, 2000; Loenen et al., 2014; Costa et al., 2017). The motif comprises two specific 3-4 bp long sequences, separated by a 5-8 bp long, non-specific, spacer sequence (Loenen et al., 2014; Costa et al., 2017; Atack et al., 2018). Since individual TRDs can shuffle between different HsdS proteins through various rearrangements between *hsdS* alleles, an extensive variety of target recognition motifs exists (Furuta et al., 2011; Loenen et al., 2014; Cooper et al., 2017; Atack et al., 2020).

In contrast to the polycistronic organization of type I systems, type II systems mostly include two separate enzymes: a methyltransferase, that targets a specific base in a specific sequence and a restriction endonuclease that cleaves DNA either at a defined site within or by the recognition sequence. Recognition motifs of type II systems are usually 4-8 bp in length and palindromic (Pingoud et al., 2014). A particular representative of the class are type IIG systems, in which the restriction enzyme and methyltransferase are transcribed from a single gene to form a contiguous polypeptide (Pingoud et al., 2014).

Type III systems are heterooligomeric complexes consisting of a methyltransferase that also determines sequence specificity (*mod*) and an endonuclease (*res*), that carries out restriction cleavage near an unmethylated recognition sequence when bound to the Mod subunit (Rao et al., 2014). Type III systems usually recognize short (5-6 bp), asymmetric motifs and have been reported to occur only rarely in staphylococci such as *S. aureus* (Sadykov, 2016; Lee et al., 2019).

Type IV systems are anti-methylation systems and only composed of one to two endonucleases. They distinguish themselves from type I to III systems as they are not associated with a respective methyltransferase. Hereby, type IV restriction enzymes solely cleave DNA harboring a particular type of methylation with a loose sequence specificity (Loenen and Raleigh, 2014). They are further thought to defend genomes against competing genome methylation systems by cell suicide (Fukuda et al., 2008).

RM systems are also relevant in respect to the genetic manipulability of cloning hosts. Particularly wildtype strains often possess strong barriers to incoming, exogenous DNA and are therefore much harder to transform compared to laboratory strains. An approach to overcome the hosts RM systems is plasmid artificial modification (PAM). PAM mimics the host target strains methylation profile by pre-passaging plasmid DNA through modified *E. coli* strains and has been shown to increase the transformation efficiency of many bacteria (Deng et al., 2000; Suzuki and Yasui, 2011; Monk et al., 2015).

The fact that methylation patterns not only serve as barrier to invading DNA but also play an important role in the context of epigenetics, should not go unmentioned here. There is a growing number of studies addressing the influence of DNA methylation mediated by the methyltransferases of RM systems on the regulation of important cellular mechanisms including replication control, the expression of virulence factors and phenotypes such as biofilm formation and host colonization as well as the phase-variable expression of genes which in turn enables cells to switch flexibly between different physiological states (Furuta et al., 2014; Atack et al., 2018, 2020; Nye et al., 2020; Yano et al., 2020; Oliveira and Fang, 2021).

The methylome of more well-known members of the genus *Staphylococcus* such as *S. aureus* and *S. epidermidis* has been extensively characterized in the past (Sadykov, 2016; Lee et al., 2019). Only little is known about the less well studied species such as *S. xylosus*, for which the presence of RM systems has only been named sporadically in a few studies (Harrison et al., 2013) but has never been characterized in detail. In this study we determined the methylome, thus all methyl-modified DNA sequences in selected *S. xylosus* strains using single molecule real-time (SMRT) sequencing in order to obtain more information about methylation patterns and the presence of RM systems within the species.

## 2. Materials and methods

### 2.1. Bacterial strains, growth, reagents

All bacterial strains, oligonucleotides and plasmids used in this study are listed in Supplementary Table 1. *Escherichia coli* and *Staphylococcus sp*. were routinely cultured at 37°C, 200 rpm in Lysogeny Broth (LB, tryptone 10 g/l, yeast extract 5 g/l, NaCl 5 g/l) and Trypticase soy broth (TSB, casein peptone 15 g/L, soy peptone 15 g/L, yeast extract 3 g/L), respectively, unless required and stated otherwise. For the respective agar plates, the liquid media were solidified with 1.5% agar (Carl Roth). All antibiotics were purchased from Carl Roth and used at the following concentrations: ampicillin (100 μg/ml), kanamycin (20 μg/ml). Oligonucleotides were obtained from Eurofins Genomics, Germany. Restriction enzymes, Gibson assembly mix, T4 DNA ligase as well as PCR components (Q5 high fidelity PCR kit) were obtained from New England Biolabs (NEB). For plasmid isolation, DNA gel extraction and PCR product purification, the NEB Monarch Plasmid Miniprep, DNA gel extraction and PCR and DNA Cleanup kits were used, respectively.

### 2.2. Transformation

Transformation of *E. coli* strains was performed by washing *E. coli* cells electrocompetent using standard protocols (Wood, 1983). Basically, 100 ml of overnight culture was harvested during mid-exponential phase (OD_600_ 0.5-0.7), placed on ice for 10 min and centrifuged at 5,000 x g, 4°C for 10 min. The supernatant was poured off and the pellet was resuspended in 100 ml of 10% glycerol. Centrifugation and resuspension steps were repeated twice more with decelerating volumes of resuspension buffer and cells were finally resuspended with 500 μl of 10% glycerol. Transformation of *E. coli* cells by electroporation was performed in a 0.1 cm cuvette (Gene pulser MicroPulser cuvette) at 1.8 kV (MicroPulser electroporator, Bio-Rad Laboratories).

### 2.3. Expression of type I and type II modification enzymes in *E. coli*

*Staphylococcus xylosus* methyltransferases were heterologously expressed in *E. coli*. The respective genes were integrated into *E. coli* strain K12 DC10B at site-specific locations (*att*B sites) of the chromosome, in a single cloning and chromosomal integration step (St-Pierre et al., 2013). We hoped that the expression of modification genes from the chromosome rather than multicopy plasmid, would result in less metabolic burden for the cell, a stable expression and subsequent complete modification (Englaender et al., 2017). The applied method is based on bacteriophage integrases mediating site-specific insertions of the genes of interest into prokaryotic chromosomes (*att*B sites). Within this study, the integrases of coliphages λ (pOSIP-KL) and 186 (pOSIP-KO) were used. The type II methyltransferase of *S. xylosus* TMW 2.1324 was amplified using primers PN25_MT_F and RS_MT_R at first, followed by a subsequent PCR reaction complemented with the dimerized oligonucleotides of promoter P_*N*25_ and primers PN25_MT_F and RS_MT_R. The promoter-gene construct was excised from an agarose gel, purified, and ligated into the linearized (*Sac*I/*Pst*I) vector pOSIP-KL. The different variants of type I systems of TMW 2.1023 and TMW 2.1324 (*hsdSMS*/*hsdMS*/*hsdMS*_*tr*_) were ligated into vector pOSIP-KO (*Kpn*I/*Sph*I) in the same way, using primer pairs PN25_*hsd*SMS_F/PN25_*hsdMS*_F and RS_*hsdS*_R/RS_*hsdS*_tr_R at first, followed by overamplification with RS_PN25_F and RS_*hsdS*_R/RS_*hsdS*_tr_R, respectively. Integration of the pBla-MTase construct was performed by amplifying the promoter from plasmid pE-Flp using primers vec_pBla_1F and Bla_Mtase_1R and the methyltransferase of TMW 2.1324 using Bla_Mtase_2F and Mtase_186_2R with subsequent Gibson assembly of all PCR products into the linearized vector pOSIP-KO (*Kpn*I/*Pst*I).

Assembled vectors were transformed into *E. coli* by electroporation, and FLP-mediated excision of the backbone was achieved by transforming cells with plasmid pE-FLP. Integration, screening for successful transformants, excision and final screening for successful integrants were performed according to the step-by-step protocol provided by Cui and Shearwin (2017).

### 2.4. Real-time quantitative reverse transcription PCR

For RT-qPCR experiments, RNA was isolated from the *E. coli* CM strains at first in three biological replicates each. Therefore, 3 ml of liquid culture were harvested during early exponential phase and RNA was extracted using the Monarch^®^ Total RNA Miniprep Kit (NEB) according to the manufacturer’s instructions. As recommended in the NEB protocol, two DNase I digestions were performed on all samples to remove residual gDNA, namely a one-column treatment as well as an in-tube treatment after purification. In-tube digestion was performed by incubating 1.35 μg of RNA at 25°C for 5 min with 2.73 Kunitz units DNAse I and 0.1x RDD buffer from the RNase-Free DNase Set (QIAgen) in a total volume of 40 μl. DNAse I was subsequently inactivated by adding 5 μl of a 25 mM EDTA solution and incubation at 75°C for 5 min. Complementary DNA (cDNA) was generated from 180 ng DNAse-treated RNA from each sample using the QuantiTect Reverse Transcription Kit (QIAgen) according to the manufacturer’s protocol. Additionally, a no reverse transcriptase (NRT) was generated for each sample. After reverse transcription, the cDNA was diluted 1:12 with nuclease-free water (Omega Bio-tek). qPCR was performed on a CFX384 Touch Real-Time PCR Detection System (Bio-Rad Laboratories, Inc.) using 6 ng of cDNA, the Luna Universal qPCR Master Mix (NEB) and the primer pairs shown in Supplementary Table 2. Cycling parameters were set to 95°C (1 min), 40 cycles of 95°C (15 s) and 60°C (30 s with plate read on SYBR channel) each, and a melt curve from 60 to 95°C with an increment of 0.5°C per 5 s and plate read on SYBR channel after each increment.

### 2.5. SMRT sequencing

Single molecule real-time (SMRT) sequencing was performed to identify modified bases of *S. xylosus* and genetically modified *E. coli* strains (Clark et al., 2012). DNA isolation was performed using the E.Z.N.A. Bacterial DNA-kit (Omega Bio-tek) according to the manufacturers instruction, except that lysostaphin (0.5 mg/ml) was included into the lysis buffer of the staphylococcal samples to weaken the cell wall. Library construction and sequencing (PacBio RS II) of *S. xylosus* followed the protocol described by Schiffer et al. (2019). *E. coli* sequencing was performed on a PacBio Sequel instrument (SMRT cell 1M), partly at the functional genomics center Zurich (ETH Zürich), and partly at the research unit for environmental genomics Munich (Helmholtz Zentrum München). Therefore, the Sequel^®^ Binding Kit 3.0 (Pacific Biosciences of California) was used and the libraries were size-selected to around 6 to 7 kb. SMRT Analysis version 7.0 (Pacific Biosciences) was used for assembly (HGAP4), base modification and motif analysis of *S. xylosus*, SMRT Link version 10.1 for assembly, base modification and motif analysis of *E. coli*. For *S. xylosus* the assembled genomes were used as their own reference, while for *E. coli*, the assembly of strain DH10B available on NCBI (NC_010473) was used as a reference.

### 2.6. Bioinformatic analysis and data availability

Sequence alignments were made using CLC main workbench 8.1.4^1^ with the built-in Clustal Omega plugin and subsequent construction of pairwise comparison matrices and phylogenetic trees (neighbor-joining). Blasting against two databases [NCBI’s conserved domain database (Marchler-Bauer et al., 2015) and the restriction enzyme database REBASE (Roberts and Macelis, 2001)] was used to confirm the affiliation of the identified enzymes to one of the restriction modification families, to identify enzymatic domains and to search for RM systems with the same DNA target sequence. The Blast Diagnostic Gene finder tool (BADGE) was used for comparative genomics in order to match the corresponding RM genes and modification patterns (Behr et al., 2016). The online available NCBI blastn and blastp tools were used to search for RM components besides the ones already annotated. The protein fold recognition server PHYRE^2^ (Kelley et al., 2015) helped in predicting secondary structure conformation of the identified polypeptides. In a previous study, a full proteome dataset was generated for *S. xylosus* TMW 2.1023 and TMW 2.1523 (Schiffer et al., 2021), which was taken into account in this study to verify the expression of single genes (Supplementary Table 3). The dataset is available under the identifier PXD029728 at the ProteomeXchange Consortium via the PRIDE partner repository (Perez-Riverol et al., 2019). All *S. xylosus* genome sequences are deposited at GenBank under the accession numbers provided in Supplementary Table 1. For *in silico* analysis, additionally available genomes on the NCBI server that were sequenced using PacBio technology were included into the analysis. The respective accession numbers are also included in Supplementary Table 1. The assembled sequences of the *E. coli* CM strains as well as the base modification analysis outputs were submitted to GenBank, too.

## 3. Results

### 3.1. Analyzing the methylome of *S. xylosus*

In order to better understand the presence of active RM systems in *S. xylosus*, we determined the DNA methylation profile of selected *S. xylosus* strains using PacBio SMRT sequencing technology and further explored the occurrence of restriction modification systems by detailed bioinformatic analysis of the genomes. Hereby, we were able to assign the respective modification and restriction enzymes to the identified methylated DNA sequences with a high degree of certainty as mostly not more than one respective open reading frame was available for choice. Table 1 provides an overview of identified RM systems and the corresponding modification patterns. Supplementary Table 4 displays the full base modification output of the sequenced strains. Six other PacBio sequenced strains of *S. xylosus* listed on REBASE were included into the overview to provide a more comprehensive picture of the prevalence of RM systems within the species.

**TABLE 1:** Overview of present restriction modification systems in selected *S. xylosus* strains and the respective base modification motifs derived from SMRT sequencing.

*S. xylosus*	Locustag	Location	Annotation	Length (nt)	Class	Assigned motif
TMW 2.1023	JGY91_01640	chromosome	Type I restriction modification subunit M	198_*trunc.*	I	None
	JGY91_13160	plasmid	Type I restriction endonuclease subunit S	1170	I	TC**A**N_6_CTC/G**A**GN_6_TGA
	JGY91_13165	plasmid	Type I restriction modification system subunit M	1557	I	
	JGY91_13170	plasmid	Type I restriction endonuclease subunit S	576	I	
	JGY91_13175	plasmid	Type I restriction endonuclease subunit R	2787	I	
	JGY91_01725	chromosome	DUF3578 domain-containing protein_McrBP	2028	IV	
	JGY91_01730	chromosome	Hypothetical protein_McrCP	1323	IV	
TMW 2.1324	JGY90_00145	chromosome	*Alw*I family type II restriction endonuclease	2121	II	GC**A**TC/G**A**TGC
	JGY90_00150	chromosome	DNA-(adenine-N6)-methyltransferase	2127	II	
	JGY90_14115	plasmid	Type I restriction endonuclease subunit S	1185	I	**A**CCN_5_RTGT/AC**A**YN_5_GGT
	JGY90_14120	plasmid	Type I restriction modification subunit M	1557	I	
	JGY90_14125	plasmid	Type I restriction endonuclease subunit S	576	I	
	JGY90_14130	plasmid	Type I restriction endonuclease subunit R	2787	I	
TMW 2.1521	JGY89_12325	chromosome	DEAD/DEAH box helicase	4737	II G	GGGTN**A**
	JGY89_12080	chromosome	Type I restriction modification subunit M	198_*trunc.*	I	
	JGY89_11995	chromosome	DUF3578 domain-containing protein_McrBP	2028	IV	
	JGY89_11990	chromosome	Hypothetical protein_McrCP	1323	IV	
TMW 2.1523	JGY88_00145	chromosome	DEAD/DEAH box helicase	4728	II G	GGGTN**A**
TMW 2.1602	None found					CAC**C**G
TMW 2.1693	LHJ66_02060	chromosome	Type I restriction modification subunit M	1515	I	G**A**CN_5_TGT/AC**A**N_5_GTC
	LHJ66_02065	chromosome	Type I restriction endonuclease subunit S	1215	I	
	LHJ66_02070	chromosome	Type I restriction endonuclease subunit R	3123	I	
	LHJ66_02820	chromosome	DNA cytosine methyltransferase	1287	II	
	LHJ66_13490	plasmid?	Site-specific DNA methyltransferase	2001	III	GCTC**A**
	LHJ66_13495	plasmid?	DEAD/DEAH box helicase family protein	2700	III	
TMW 2.1704	LHJ68_05155	chromosome	DNA cytosine methyltransferase	1047	II	
	LHJ68_05160	chromosome	DNA cytosine methyltransferase	1080	II	
	LHJ68_05170	chromosome	DNA cytosine methyltransferase	1188	II	
TMW 2.1780	LHJ67_11845	chromosome	DEAD_DEAH box helicase family protein	4737	II G	GGGTN**A**
2	DWB98_00235	chromosome	Type I restriction modification subunit M	1464	I	
	DWB98_00240	chromosome	Type I restriction endonuclease subunit S	1164	I	
	DWB98_00245	chromosome	Type I restriction endonuclease subunit R	3354	I	
DMSX03	DMSX03_RS00135	chromosome	Site-specific DNA-methyltransferase	1923	III	
	DMSX03_RS00140	chromosome	Restriction endonuclease	2967	III	
	DMSX03_RS00200	chromosome	DUF3578 domain-containing protein_McrBP	2034	IV	
	DMSX03_RS00205	chromosome	Hypothetical protein_McrCP	1323	IV	
HKUOPL8	BE24_RS11845	chromosome	Type I restriction modification subunit M	1515	I	
	BE24_RS11850	chromosome	Type I restriction endonuclease subunit S	1251	I	
	BE24_RS11855	chromosome	Type I restriction endonuclease subunit R	3123	I	
	BE24_RS11615	chromosome	Cytosine methyltransferase	1080	II	CCCGT
	BE24_RS11620	chromosome	DNA methyltransferase	1047	II	CCCGT
	BE24_RS13495	chromosome	AAA family ATPase	1473	II	
	BE24_RS11635	chromosome	LlaJI family restriction endonuclease	1122	II	
	BE24_RS05200	chromosome	DNA methyltransferase (C5)	957	II	
S170	AWC37_RS12155	chromosome	Type I restriction modification subunit M	1515	I	
	AWC37_RS12160	chromosome	Type I restriction endonuclease subunit S	1263	I	
	AWC37_RS12165	chromosome	Type I restriction endonuclease subunit R	3123	I	
	AWC37_RS12130	chromosome	DUF3578 domain-containing protein_McrBP	2034	IV	
	AWC37_RS12125	chromosome	Hypothetical protein_McrCP	1323	IV	
SMQ-121	SXYLSMQ121_ RS00165	chromosome	Type I restriction modification subunit M	1515	I	C**A**CN_4_RTTG/ GTGN_4_Y**A**AC
	SXYLSMQ121_ RS00160	chromosome	Type I restriction endonuclease subunit S	1266	I	
	SXYLSMQ121_ RS00155	chromosome	Type I restriction endonuclease subunit R	3123	I	
	SXYLSMQ121_ RS00195	chromosome	DUF3578 domain-containing protein_McrBP	2034	IV	
	SXYLSMQ121_ RS00195	chromosome	Hypothetical protein_McrCP	1317	IV	
C2a	SXYL_RS00155	chromosome	Restriction endonuclease subunit R	423	I	
	SXYL_RS00165	chromosome	DUF3578 domain-containing protein_McrBP	2034	IV	
	SXYL_RS00170	chromosome	Hypothetical protein_McrCP	1317	IV	

Further indicated are location on RM system class (I-IV). Bases in bold correspond to the methylation sites if known.

In terms of type I RM systems, out of the 14 strains analyzed, seven carry a complete type I RM system in their genome (presence of *hsdM*, *hsdS*, and *hsdR*). None of the *S. xylosus* strains harbors more than one type I RM system, nor any orphan *hsdS* genes. Notably we found two different types of operons, five strains harbor a chromosomally encoded type I system organized as a contiguous three-gene (*hsdMSR*) operon while two strains (TMW 2.1023 and 2.1324) carry a plasmid encoded four-gene (*hsdRSMS*) operon.

Despite type I base modifications, other motifs such as GCATC, a common type II motif with more than 600 hits on REBASE, present across a wide range of species such as *Mycoplasma bovis, Mannheimia haemolytica* and *Streptococcus pneuomoniae* were also identified in strains such as TMW 2.1324. Interestingly, three *S. xylosus* strains (TMW 2.1521, 2.1523 and 2.1780) possess a type IIG system, which comprises a single enzyme, mediating methyltransferase as well as endonuclease activity. The detected type IIG systems are all associated with the same modification pattern (GGGTNA) and gene sequence analysis did not reveal any frameshifts in the gene sequences. Furthermore, data derived from whole proteome analysis (Schiffer et al., 2021) confirmed the expression of a functional type IIG enzyme of the expected amino acid sequence in TMW 2.1523 (Supplementary Table 3).

Blasting of methyltransferase genes against the REBASE database also revealed the presence of type III systems in strains TMW 2.1693 (LHJ66_13490-95) and DMSX03 (DMSX03_RS00135-40). The only strain, for which no respective methyltransferase could be assigned to the determined modification pattern is TMW 2.1602. According to its kinetic signature during PacBio sequencing, the strain modifies the motif CACCG, which could be a type II or type III motif. Nevertheless, using comparative genomics, no strain-specific methyltransferases or endonucleases were identifiable for this strain, nor did the online available Restriction-ModificationFinder (Roer et al., 2016) identify any RM systems in the genome of TMW 2.1602. The motif is not listed on REBASE either, therefore no further conclusions on this strain’s methylation can be drawn at the time. Genome analysis additionally revealed, that some *S. xylosus* strains, namely TMW 2.1693, TMW 2.1704, HKUOPL8 and S04010, encode cytosine methyltransferases, probably mediating 5-methylcytosine (m5C) modification. However, checking whether these enzymes are active or which motifs they modify is difficult since it is challenging to use SMRT sequencing technology to distinguish m5C from cytosine (Clark et al., 2012; Loenen et al., 2014).

Lastly, endonucleases belonging to the type IV RM family were identified in strains TMW 2.1023, TMW 2.1521, DMSX03, S170, SMQ-121 and C2a (Table 1). The identified type IV systems encompass two endonucleases which are encoded in tandem with overlapping reading frames on the chromosome. The type IV system includes a DUF3578 domain and resembles, in its sequence structure, the well-characterized, two subunits containing McrBC 5-methylcytosine restriction system of *Escherichia coli* K-12.

### 3.2. *In silico* analysis of type I RM systems reveals a new operon structure

Type I RM systems were identified in seven out of the 14 analyzed *S. xylosus* strains, making their presence within the species non-ubiquitous. While a common gene order of the *hsd* operon (*hsdRSM*/*hsdMSR*) is chromosomally encoded in strains TMW 2.1693, 2, HKUOPL8, S170 and SMQ-121, an unusual gene arrangement (*hsdRSMS*) was found on the plasmids harbored by TMW 2.1023 and TMW 2.1324, with two genes of different lengths, both annotated as *hsdS* surrounding the methyltransferase (*hsdM*). To confirm that none of the *hsdS* subunits is truncated, the proteomic dataset obtained from a previous study was consulted again, confirming the expression of both *hsdS* subunits in TMW 2.1023 (Supplementary Table 3). The first *hsdS* (*hsdS*_short) subunit of the system is 191 aa in length and the second one (*hsdS*_long) around 390 - 400 aa. Furthermore, the 3′ end of *hsdM* overlaps by 8 bp the 5′ end of the second *hsdS*_long subunit. Because of the organization of the ORFs directly to one another (*hsdS*-*hsdM*-*hsdS*), with the *hsdM*-*hsdS* 8 bp overlap and a conserved Shine-Dalgarno binding site preceding each ORF, it can be assumed that the genes are co-transcribed under the control of a single promoter in both *S. xylosus* strains. We also note that putative promoter sequences (canonical consensus σ70 -35/-10) are present in front of *hsdR* and *hsdS*_short. Polycistronic gene organization facilitates enhanced regulatory control through translational coupling between genes of related functional partners to control subunit stoichiometry and was previously described for type I restriction systems (Deng et al., 2000; Roberts et al., 2012). Interestingly, *hsdRSMS* systems are part of a large plasmid in both *S. xylosus* strains. Blasting the individual genes of the operon reveals that the system is located on at least eleven further staphylococcal plasmids (*hsdRSMS*_PL_) as well as it was found that some staphylococcal species also carry the four gene operon on their chromosome (*hsdRSMS*_CHRM_). Yet, it appears as if *hsdRSMS*_CHRM_ is mostly encoded on mobile genetic elements (MGEs) on the chromosome, often being part of staphylococcal cassette chromosome (SCC) genomic islands. Also, recombinases are frequently encoded just a few genes upstream or downstream of the operon. Table 2 lists all strains encoding the four gene *hsdRSMS* operon on a plasmid as well as a selection of strains that have the operon encoded on their chromosome.

**TABLE 2:** Overview of organisms harboring the *hsdRSMS* systeme either on a plasmid (pL) or on the chromosome (chrm).

Organism	Strain	pL/chrm	Located on MGE	Accession (Genbank)
*Staphylococcus xylosus*	TMW 2.1023	pL1	-	JAEMUG010000002
*Staphylococcus xylosus*	TMW 2.1324	pL1	-	CP066727.1
*Staphylococcus aureus*	SA01	pSA01-tet	-	CP053076.1
*Staphylococcus aureus*	55–100–016	pL1	-	CP076840.1
*Staphylococcus aureus*	UP_966	pL1	-	CP047831.1
*Staphylococcus aureus*	HUV05	pHUV05–03	-	CP007679.1
*Staphylococcus equorum*	C2014	pC2014–2	-	CP013716.1
*Staphylococcus hominis*	FDAARGOS_762	pL3	-	CP054008.1
*Staphylococcus nepalensis*	JS1	pSNJS101	-	CP017461.1
*Staphylococcus pseudoxylosus*	14AME19	p14AME19–2	-	CP068714.1
*Staphylococcus saprophyticus*	UTI-045	pUTI-045-1	-	CP054832.1
*Staphylococcus aureus*	45394F	chrm	SCC	GU122149.1
*Staphylococcus aureus*	ER02693.3	chrm	recombinase	CP030605.1
*Staphylococcus caprae*	SY333	chrm		CP051643.1
*Staphylococcus carnosus*	FDAARGOS_1147	chrm	recombinase	CP068079.1
*Staphylococcus condimenti*	FDAARGOS_1205	chrm	recombinase x 2	CP069567.1
*Staphylococcus epidermidis*	FDAARGOS_161	chrm	transposase	CP014132.1
*Staphylococcus equorum*	FDAARGOS_1149	chrm	recombinase, transposase	CP068069.1
*Staphylococcus hominis*	TFGsh1	chrm	SCC	AB930126.1
*Staphylococcus saprophyticus*	UTI-042y	chrm	recombinase	CP054438.1
*Staphylococcus schleiferi*	OT1-1	chrm		CP035007.1
*Mammaliicoccus fleurettii*	FDAARGOS_682	chrm		CP046351.1

In *S. pseudoxylosus* 14AME *hsdS*_long is truncated and the *hsdRSMS* systems of *S. aureus* UP966 is disrupted by a transposon. Genes indicating a localization on a mobile genetic element (MGE), identified in the surrounding of the operon, are listed when found.

**FIGURE 1:**
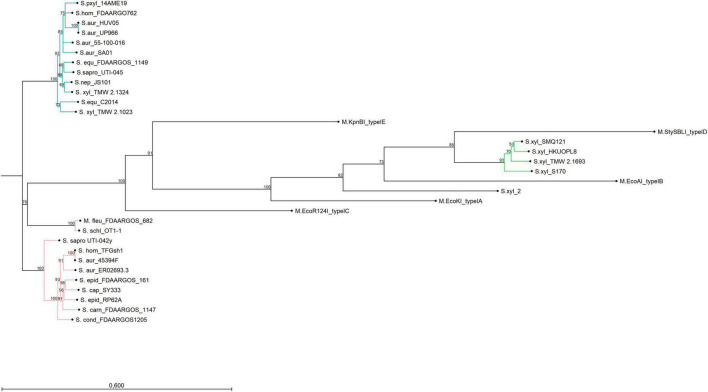
Neighbor-joining tree displaying the phylogenetic topology of *hsdM* from type I RM systems of different bacterial organisms and strains. The turquoise group represents *hsdM* genes of *hsdRSMS*_PL_ systems, the green group belongs to *S. xylosus* chromosomal *hsdMSR* systems and the group in rose encompasses *hsdM* genes of *hsdRSMS*_CHRM_ systems. The only outlier is *hsdM* of *S. equorum* FDAARGOS_1149 which is chromosomally encoded but clusters with the plasmid-based group. Reference genes of type I systems (A-E) were included into the Figure. The bar indicates 60% sequence divergence.

**FIGURE 2:**
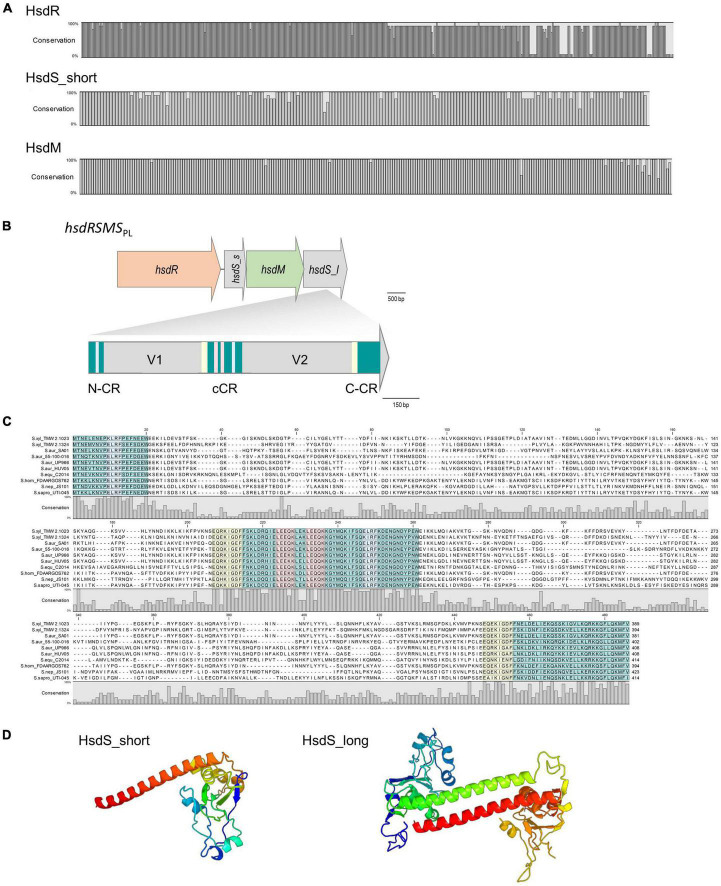
**(A)** Conservation plots based on amino acid alignments of HsdR, HsdM and *HsdS*_short derived from *hsdRSMS*_PL_ systems (11 sequences each were aligned, full alignments are shown in Supplementary Figures 4, 5). **(B)** Gene arrangement of the four genes containing *hsdRSMS* operon. *hsdS*_s = *hsdS*_short, *hsdS*_l = *hsdS*_long. HsdS_long is composed of two variable regions (V1 and V2) as well as an N-terminal (N-CR), C-terminal (C-CR) and central conserved region (cCR). All conserved regions are marked in turquoise. Other repeating sequences are marked in red, blue and yellow, respectively. Note the frameshift at the junction between coding regions: in *hsdRSMS*, *hsdM* overlaps *hsdS*_long by 8 bp **(C)**. HsdS_long subunit alignment on amino acid level of all *hsdRSMS*_PL_ systems listed in Table 2. Conservation plot shows the low conservation in the variable region as well as the repeating sections (blue, yellow, red) of the conserved (turquoise) regions. **(D)** Protein fold prediction based on PHYRE for HsdS_short and HsdS_long exemplarily for strain TMW 2.1023. The coiled coil region (red/green) displays the conserved region connecting the two TRDs which are colored in blue and yellow to orange (99.9% modeling confidence).

Alignments and gene topology analysis was performed to classify all discovered *S. xylosus* type I RM systems (three and four gene operons) into one of the five existing type I families (A-E). Percent identity and distant values for alignments of *hsdR* and *hsdM* with the reference genes of the respective families are provided in the comparison matrix of Supplementary Figure 1. Hereby, *hsdM* and *hsdR* of the *S. xylosus* three-gene *hsdMSR* operons are closest to the reference genes of family type ID RM systems (StySBLI) sharing 50% (*hsdM*) and 40% (*hsdR*) percent sequence identity, respectively. An exception is *S. xylosus* strain 2, which cannot clearly be categorized as it carries a *hsdMSR* system with identity values below 30% to any of the reference genes. Methyltransferases (*hsdM*) and endonucleases (*hsdR*) of the *hsdRSMS* four gene operon display as little as 7% identity to family IB (M.EcoAI) and ID (M.StySBLI) and a maximum of 48% (*hsdM*) and 40% (*hsdR*) identity to the type IC reference genes (EcoR124I). Interestingly, intraspecies percent identity values of *hsdM* genes, namely *hsdM* of *S. xylosus hsdRSMS* operons and *hsdM* of *S. xylosus hsdMSR* operons were all below 10%, substantiating the hypothesis that the *hsdRSMS* operon represents a differentiated system. The phylogenetic trees provided in Figure 1 (*hsdM*) and Supplementary Figure 2 (*hsdR*) further reveal that neither *hsdR* nor *hsdM* of the *hsdRSMS* operon cluster with any of the type I family reference genes. In contrast, the phylogenetic distance of the three-gene operon (*hsdMSR*) of the other *S. xylosus* strains to type ID systems is smaller and they group together. Interestingly, the trees even distinguish between the two types of *hsdRSMS* systems found, with *hsdM* and *hsdR* of *hsdRSMS*_PL_ systems showing a distinct phylogenetic distance to *hsdRSMS*_CHRM_ systems. The only exception here is *S. equorum* FDAARGOS_1149, that carries a *hsdRSMS*_CHRM_ system clustering together with the *hsdRSMS*_PL_ systems. Alignments of each gene of *hsdRSMS*_PL_ separately revealed that *hsdM*, *hsdR* and *hsdS*_short are well conserved along the entire sequence (Figure 2A). Alignments of *hsdS*_long resulted in a typical conservation plot often seen for *hsdS* subunits, with three conserved regions (N-, C -terminal, central) flanking two variable regions, each dedicated as one TRD (Figures 2B, C). No tetra amino acid repeats as previously described for type IC *hsdS* subunits (Adamczyk-Popławska et al., 2003) could be identified in the central conserved region. We did identify two short repeating stretches in the central region, though (2x LEEQK), as well as part of the central sequence is repeated in the N- and C- terminal conserved regions, respectively, (Figure 2C). Important to mention is that long and short *hsdS* subunits of one *hsdRSMS* operon don’t share any common, homologous regions. Further, the repeats found in the long *hsdS* subunits do not exist in the short ones. Taking secondary structure into consideration a typical protein fold was predicted by PHYRE (Kelley et al., 2015) for *hsdS*_long with the two TRDs connected by alpha helices/coiled coil structures in an antiparallel order (Figure 2D), whereas for *hsdS*_short, a strikingly similar structure to a halfsize *hsdS* subunit is predicted. Referring to the NCBI Conserved Domain Database (Marchler-Bauer et al., 2015), *hsdS*_long subunits consist of two TRDs and *hsdS*_short of one. However, while this result is consistent for *hsdS*_long genes as they comprise two variable regions flanked by conserved regions, it is less clear for *hsdS*_short as the entire sequence is conserved, not harboring any variable parts. Furthermore, when blasting the individual TRDs of *hsdS*_long, it yields hits on other *hsdS* subunits, emphasizing the dynamic, interallelic recombination of single TRDs between *hsdS* subunits. However, a contrary result is obtained when *hsdS*_short is blasted against the NCBI database. According to the results obtained, *hsdS*_short seems to never occur as part of a *hsdS*_long subunit. This fact clearly votes for *hsdS*_short not being a halfsize or truncated *hsdS* subunit but rather an individual gene with a specific function, not flipping and recombining with *hsdS*_long subunits. One last noteworthy fact is, that it has been reported previously that *hsdS* genes, even if they are not part of the same family, share high homology (> 50%) among their variable regions determining the TRDs, if they recognize the same nucleotide motif (Murray, 2000). According to REBASE, the type I system of numerous *E. coli* strains (e.g., NCTC9029) as well as *Anaerobiospirillum thomasii* NCTC12467 recognize the same motif as *S. xylosus* TMW 2.1023 as well as certain *S. aureus* strains (AUS0325, WBG8366, MRSA - AMRF 6, MRSA - AMRF 4, ER09113.3) and TMW 2.1324 share a type I system recognizing the same target DNA sequence. When aligning the TRDs accordingly, percent identity values of 69% (N-TRD) and 63% (C-TRD), respectively, were obtained for TMW 2.1324 and the HsdS subunits of the *S. aureus* strains, compared to 20% amino acid sequence identity when aligning the TMW 2.1023 TRDs with HsdS of the *S. aureus* strains. In contrast, the TRDs of TMW 2.1023 did not show any significant similarity to neither the HsdS subunits of *A. thomasii* NCTC12467 nor *E. coli* NCTC9029, despite recognizing the same sequence motif (percent identity values around 21%).

### 3.3. Expression of modification systems in *E. coli*

In order to confirm the specificity of selected methyltransferases and to characterize the function of the newly detected *hsdRSMS* system in more detail, methyltransferases and specificity units were heterologously expressed in *E. coli*. As expression host functioned *E. coli* DC10B, a *dcm* - negative K12 derivate, unable to methylate cytosine. Modification enzymes were integrated into and expressed from the chromosome as the expression on a plasmid has previously been associated with instability and inadequate base modification (Lee et al., 2019). To determine the most suitable promoter, which provides a complete methylation of the target DNA but does not pose a too heavy burden for the cell, the less complex type II system of TMW 2.1324 was used as a test system. Therefore, the corresponding type II methyltransferase gene (motif GATGC/GCATC) was integrated into the *E. coli* chromosome transcribed from two different constitutive promoters, the β-lactamase promoter P_bla_ as well as the T5 coliphage promoter P_N25_. Subsequent sequencing and base modification analysis revealed that only 42-76% of the existing motifs were modified when P_bla_ was used (Table 3, *E. coli* CM2) compared to 99.7% modification when the gene is under the control of P_N25_ (*E. coli* CM93). The difference in methylation propensity is also clear when digesting isolated plasmid DNA from these two strains with *Sfa*NI. *Sfa*NI recognizes the same motif as the type II system of TMW 2.1324, thus, proper modification by the respective methyltransferase should protect the plasmid from restriction. While a complete restriction digest was visible on the gel when plasmid of *E. coli* DC10B was used, an incomplete digest was detectable for plasmids isolated from *E. coli* CM2 (P_bla_) and no digestion was visible when plasmid was isolated from *E. coli* CM93 (P_N25_) (Supplementary Figure 3).

**TABLE 3:** Base modification analysis of heterologously expressed *S. xylosus* methyltransferases in *E. coli* using different gene combinations and promoters.

Strain	Expressed MT (*att*-site)	Promoter	MotifString	CenterPos	Mod	Fraction	nDetected	nGenome	MeanScore	MeanCov	Expressed typeI construct
CM56	*hsdSMS*_023 (186-2)	P_N25_	GATC^1^	2	m6A	1.00	37673	37676	1408	632.5	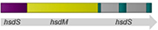
			TCANNNNNNCTC	3	m6A	0.94	1118	1192	880	632.6	
			GAGNNNNNNTGA	2	m6A	0.93	1109	1192	798	632.6	
CM57	*hsdMS*_023 (186-2)	P_N25_	GATC^1^	2	m6A	1.00	38560	38594	289	185.3	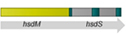
			HTCANNNNNACTCD	4	m6A	0.49	99	203	172	187.8	
			HGAGNRNNNNTGAD	3	m6A	0.32	129	399	158	187.0	
			TCABTNNBNCTC	3	m6A	0.44	89	202	168	185.8	
			GAGNNNNAVTGAND	2	m6A	0.36	72	198	162	185.9	
CM5	*hsdSMS*_324 (186-1)	P_N25_	GATC^1^	2	m6A	1.00	38557	38594	277	176.8	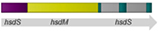
			ACAYNNNNNGGT	3	m6A	0.98	652	664	231	176.9	
			ACCNNNNNRTGT	1	m6A	0.98	649	664	219	176.6	
CM13	*hsdSMS*_MT_324 (λ, 186-1)	P_N25_	GATC^1^	2	m6A	1.00	38555	38594	243	152.8	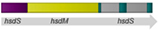
			GATGC^2^	2	m6A	1.00	14345	14382	224	152.5	
			ACAYNNNNNGGT	3	m6A	0.98	648	664	203	152.1	
			ACCNNNNNRTGT	1	m6A	0.97	645	664	194	151.4	
			GCNBGGATGC	2	m4C	0.17	32	189	134	148.3	
CM19	*hsdMS*_MT_324 (186-1)	P_N25_	GATC^1^	2	m6A	1.00	38515	38594	202	124.6	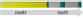
			GATGC	2	m6A	0.99	14279	14382	187	124.5	
			GCATC	3	m6A	1.00	14333	14382	185	124.5	
			ANNNNNNHNGCATGCV	12	m6A	0.19	36	189	149	128.5	
CM30	*hsdMS*_*tr*__MT_324 (λ, 186-2)	P_N25_	GATC^1^	2	m6A	0.98	37771	38594	206	118.2	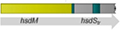
			GATGC	2	m6A	0.97	13916	14382	193	118.7	
			GCATC	3	m6A	0.97	13899	14382	188	118.6	
CM93	MT_324 (λ)	P_N25_	GATC^1^	2	m6A	1.00	38524	38594	251	147.7	
			GATGC	2	m6A	1.00	14333	14382	233	148.0	
			GCATC	3	m6A	1.00	14340	14382	229	147.8	
CM2	MT_324 (186-1)	P_bla_	GATC^1^	2	m6A	1.00	38508	38594	256	150.4	
			GATGC	2	m6A	0.76	10930	14382	156	156.1	
			GCATC	3	m6A	0.42	6025	14382	134	164.2	

*hsdS*_short is colored in violet, *hsdM* in yellow, *hsdS*_long in turquoise (conserved regions) and gray (variable regions). The *att*-sites into which the genes were integrated are indicated in brackets. Motifs with low fraction scores such as GCNBGGATGC are specific cases of prevailing motifs (GATGC) and can therefore be considered as PacBio noise.

^1^GATC is an *E. coli* motif, controlled by *E. coli*’s intrinsic adenine methyltransferase *dam*.

^2^In CM13, a mutation of the type II methyltransferase occurred due to the integration of an insertion element (IS1 family transposase). This resulted in hemi-methylation of the DNA probably due to the truncation of one of the two TRDs of the MTase by the insertion of the IS element (see Supplementary Figure 6).

Expressing from promoter P_N25_, modification genes of the newly detected four gene type I operons were integrated into the chromosome of *E. coli*, namely the full *hsdSMS* system of *S. xylosus* strains TMW 2.1023 (*E. coli*_CM56) and 2.1324 (*E. coli* CM5 and CM13), respectively, as well as *hsdMS* only, neglecting *hsdS*_short (*E. coli* CM57 and CM19). Results are listed in Table 3. In both cases, the expected motif was only properly methylated when the full *hsdSMS* operon was expressed in *E. coli* (CM56, CM13/CM5). On the contrary, if *hsdS*_short was missing, random motifs and/or modified motifs with a changed specificity and low fraction scores appeared (CM19, CM57). At this point, it should also be mentioned that attempts to complement *hsdS*_short in CM19 and CM57 to restore the full methylation specificity of the type I system remained unsuccessful. Expression of *hsdS*_short under the control of PN25 from the common *E. coli* expression vector pET3a resulted in toxic effects on the cell. Only cells in which IS elements had inserted into the construct or the plasmid had recombined so that expression of the protein no longer occurred, were able to survive. We could not fully elucidate the exact cause of the toxic effect of *hsdS*_short in this study, but the data reconfirm how profoundly an artificial methylation pattern can interfere with the regulation of essential processes in the cell.

To confirm that the observed differences in methylation are not caused by differences in gene expression, RT-qPCR was employed to quantify transcription levels. The data has been normalized to three housekeeping reference genes (*cysG*, *hcaT*, and *recA*) to be able to compare inter-strain mRNA transcription levels and to control for errors between samples. The relative gene expression between the different *E. coli* CM strains (fold change) was calculated from the mean C_q_ values between all biological replicates using the 2 ΔΔC_q_ method. The normalized mean quantification values clearly showed that all heterologously expressed constructs (*hsdSMS*, *hsdMS*) were transcribed in the respective CM strains in similar amounts (Supplementary Table 5). Precisely, the expression levels of *hsdM* and *hsdS* of the *hsdMS* constructs in comparison with the expression levels of the corresponding *hsdSMS* constructs are within a range of 0.36-fold to a maximum of 1.23-fold expression for *hsdM* and between 0.52 and 1.06 for the respective *hsdS* genes (Supplementary Table 6). Due to the small differences in expression levels, we conclude that the differences in methylation are not due to a reduced expression of the genes. Yet, it should be mentioned at this point that no data on translation are available, as we did not determine the proteins in this case.

The function of *hsdS*_long and the influence of the presence of *hsdS*_short was further tested in another experiment. For type IC RM systems, deletion of one half of the specificity unit (*hsdS)* does not impair the function of the system, it only results in a change of the TRM to a symmetric, palindrome specificity (Abadjieva et al., 1993; MacWilliams and Bickle, 1996). Attempts to reproduce these results by expressing only the N-terminal part (TRD1 and central conserved region) of TMW 2.1324 *hsdS*_long in *E. coli* (CM30) in the absence of *hsdS*_short resulted in no modification patterns at all.

## 4. Discussion

In this study we describe the prevalence of RM systems in *Staphylococcus xylosus*. We found, that *S. xylosus* harbors a variety of RM systems that are distinctly different from those of other well-studied staphylococci, such as *S. aureus* and *S. epidermidis*. Among the most prevailing differences is the presence of at most one type I system in *S. xylosus*, which is always arranged in a contiguous operon. On the contrary, up to three functioning type I systems per isolate have been reported for *S. epidermidis* strains, yet at the same time around 38% of *S. epidermidis* genomes were found to contain no functional type I RM system at all (Lee et al., 2019). Studies on *S. aureus* report *hsdR* to be spatially distant on the chromosome from *hsdMS* and that members of the species harbor at least one and up to three functional systems per strain (Monk et al., 2015; Lee et al., 2019). Furthermore, type IIG systems have been reported as inactive in *S. aureus* and type III systems as rarely present (Jones et al., 2015; Sadykov, 2016). In *S. xylosus* on the contrary, both systems appear to be active and more common as proven in this study by methylome, bioinformatic and proteomic analysis. In terms of type IV systems, the *S. aureus* subsp. *aureus* USA300 type IV system SauUSI has been deeply studied, consisting of one endonuclease polypeptide recognizing and cleaving the cytosine modified motif SCNGS (Xu et al., 2011). Again, *S. xylosus* differs here, as all identified type IV systems appear to consist of two subunits rather than one single restriction endonuclease.

Special emphasis of this work was laid on type I RM systems, which we could identify in seven out of the 14 investigated *S. xylosus* strains. Among the type I-positive *S. xylosus* isolates, we found two different types of *hsd*-operons. Firstly, chromosomally encoded three-gene *hsdMSR* operons, resembling in their gene and sequence structure other type I systems described for staphylococci and also other Gram-positive bacteria before (Lee et al., 2019; Reva et al., 2019; Finn et al., 2021). Namely, they are arranged in an operon like structure, in the order of transcription, including the three typical genes, *hsdR*, *hsdM*, *hsdS*. Secondly, we identified a hitherto undescribed variant of type I systems, *hsdRSMS*. The operon shares some common features with other staphylococcal type I systems such as the localization on mobile genetic elements (MGEs) of the chromosome and on plasmids (Lee et al., 2019) as well as the usual gene arrangement with *hsdSMS* all being transcribed from a shared promoter and *hsdR* being associated with its own promoter (Murray, 2000). Yet, in contrast to other type I systems, *hsdRSMS* requires two specificity units for proper and stable base modification, a long and a short subunit. While *hsdS*_long resembles known specificity units in its composition consisting of variable regions (TRDs) flanked by conserved regions, for *hsdS*_short such typical structural features are not evident, as it is lacking any variable regions and is highly conserved among different strains methylating different motifs. This makes it unlikely that *hsdS*_short is involved in target sequence recognition. Likewise, there is no indication for *hsdS*_short being a remnant, truncated half-size *hsdS* polypeptide. Fragmented *hsdS* genes have been reported for other type IC systems [e.g., *Ngo*AV, *Eco*DXXI, *Eco*R124I (Abadjieva et al., 1993; MacWilliams and Bickle, 1996; Piekarowicz et al., 2001)] with the C-terminal domain coding for the long *hsdS* peptide usually missing, resulting in palindromic recognition motifs. Our data showed that *hsdS*_short does not exist as part of a long *hsdS* subunit though. It is functionally expressed as well as it contributes to specific base modification of non-palindromic motifs. Upon methylation of DNA, type I methyltransferases usually form a M_2_S trimer, whereas for restriction a pentamer consisting of either R_2_M_2_S_1_ or R_1_M_2_S_1_ is formed (Gao et al., 2020). One could speculate that *hsdS*_short might have a stabilizing role in these complexes, somehow promoting binding of *hsdS*_long to *hsdM* since missing *hsdS*_short resulted in DNA target motifs with a modified specificity and low modification scores. Further studies are needed to determine the exact role of *hsdS*_short during complex assembly of the newly discovered type I RM system.

Classification of type I RM systems into one of the five existing families is based on sequence similarity values of *hsdR* and *hsdM* genes, as they are usually well conserved. However, clear cutoff values have not been determined so far and values specified in the literature vary strongly. Yet in trying to find consent, one could conclude that *hsdM* and *hsdR* share usually over 70% sequence similarity when they are members of the same family and < 30% when they are part of different families (Murray, 2000; Titheradge et al., 2001; Chin et al., 2004; Cooper et al., 2017; Gao et al., 2020). *HsdR* and *hsdM* of the *hsdRSMS* system share highest percent identity values with the reference gene of type IC systems (EcoR124I), namely 40 and 48%, respectively. Thereby, they are just at the interface between classifying them into the type IC family or establishing a new family for them. Voting for classifying them into the family of type IC systems is their occurrence on plasmids and MGEs which is characteristic for many RM systems especially members of the type IC family (Kobayashi, 2001; Youell and Firman, 2008; Loenen et al., 2014). Moreover, according to Gao et al. (2020), HsdM of type IC families is composed of three domains, namely a N-terminal (aa 11 – 190), a catalytic (aa 198 – 473) and a C-terminal (aa 481 – 510) domain. HsdM of HsdRSMS_PL_ systems displays 33% protein sequence identity to the N-terminal, 44% to the catalytic and 13-23% to the C-terminal domain of type IC M.EcoR124I (data not shown). Thus, even though both methyltransferases are arranged into a similar domain structure, single domains are not reaching sequence identity values over 44%. Therefore, voting against grouping the new operon into the family of type IC systems is not just the overall comparatively low sequence homology (∼40%) of *hsdR* and *hsdM* with the respective type I reference genes but also that *hsdS*_long is lacking some important structural and functional characteristics. Most importantly, the long subunit is not able to function independently without the presence of *hsdS*_short. Additionally, type IC *hsdS* subunits usually harbor characteristic tandem tetra amino acid repeats [e.g., TAEL, LEAT, SEAL or TSEL (Adamczyk-Popławska et al., 2003)] in their central conserved region. These repeats define among others, the spacer length between the two specificity elements of the recognition motif, with two and three repeats correlating with a 6-7 bp spacer, respectively, (Abadjieva et al., 1993; Adamczyk-Popławska et al., 2003). No such tetra amino acid repeats were identified in the central conserved region of *hsdS* from the *hsdRSMS* system, though we did find two short repeating amino acid stretches in the central conserved region (2x LEEQK). However, they are separated by 3 non-specific amino acids, thus not arranged in tandem and they do not seem to influence spacer length, as both *hsdS*_long subunits investigated in this study harbor two of such repeats but the TRDs of the TMW 2.1023 motif are divided by a 6 bp spacer compared to a 5 bp spacer in the motif of TMW 2.1324.

By methylome analysis of *S. xylosus*, this study provided new insights into the diversity of RM systems encoded by the genus *Staphylococcus*. The study further revealed the presence of a new variant of type I RM system that seems to require two specificity units for specific and thorough DNA methylation. Interestingly, the occurrence of this variant is not restricted to *S. xylosus*, as it was found to be present in other staphylococcal species as well. Additional approaches such as subunit complementation tests or antibody cross reactivity assays could further define the family affiliation in the future. All in all, the results obtained from this study present another piece in the mosaic of the diversity of methylation systems in bacteria.

## Data availability statement

The datasets presented in this study can be found in online repositories. The names of the repository/repositories and accession number(s) can be found in this article/Supplementary material.

## Author contributions

CS: conceptualization, data curation, investigation, methodology, software, visualization, and writing—original draft. CG: investigation, methodology, and software. MP: supervision and methodology. RV: funding acquisition, supervision, and writing—review and editing. ME: conceptualization, investigation, methodology, supervision, and writing—review and editing. All authors contributed to the article and approved the submitted version.

## Footnotes

1
https://digitalinsights.qiagen.com/
