# Supplementary material for: Characterization of the Staphylococcus xylosus methylome reveals a new variant of type I restriction modification system in staphylococci
Source: Front Microbiol. 2023 Mar 8;14:946189. doi: 10.3389/fmicb.2023.946189 (PMC10030836; doi:10.3389/fmicb.2023.946189)
Supplement: Supplementary file 1 [file Data_Sheet_1.PDF]

Figure S1: Pairwise comparison matrix based on Clustal-O alignments of *hsdM* (A) and *hsdR* (B) genes investigated in the scope of this study. Reference genes for type I family A - E were included. Percent identity (upper) and distance (lower matrix) values are shown in green and red, respectively.

Type I restriction modification subunit *hsd*M

Group	Strain		1	2	3	4	5	6	7	8	9	10	11	12	13	14	15	16	17	18	19	20	21	22	23	24	25	26	27	28	29 typeIA	30 typeIB	31 typeIC	32 typeID	33 typeIE
*hsd*RSMS$_{PL}$	S. xyl_TMW 2.1023	1		92,74	92,42	91,27	89,27	89,27	91,91	91,71	92,61	90,82	92,10	75,72	75,98	76,11	75,59	77,14	76,11	76,36	92,23	75,72	79,13	75,92	75,79	9,22	8,60	8,64	8,60	8,72	9,42	7,79	47,79	8,12	26,02
	S. xyl_TMW 2.1324	2	0,08		95,12	93,00	91,39	91,39	91,01	93,38	96,27	93,83	94,35	77,39	76,81	76,56	76,36	77,52	76,75	76,81	94,80	77,39	80,35	76,75	76,69	9,18	8,40	8,36	8,36	8,48	9,62	7,71	48,17	7,65	26,24
	S.aur_SA01	3	0,08	0,05		95,25	92,87	92,87	89,92	94,41	93,32	93,00	93,38	76,75	76,24	75,98	75,47	76,94	76,24	76,24	93,26	76,75	80,15	76,49	76,43	8,77	8,36	8,36	8,24	8,48	9,46	7,50	47,85	7,53	26,81
	S.aur_55-100-016	4	0,09	0,07	0,05		94,03	94,03	90,82	95,57	92,74	94,93	92,68	76,49	76,24	75,66	76,11	76,81	75,98	76,56	92,68	76,49	79,70	76,36	76,24	8,81	8,36	8,40	8,28	8,56	9,46	7,83	48,36	7,41	26,69
	S.aur_UP966	5	0,12	0,09	0,07	0,06		100,00	90,62	94,86	92,04	94,48	91,46	77,14	76,88	75,98	75,98	77,71	76,62	76,69	92,04	77,14	80,35	76,36	76,24	8,85	8,20	8,20	8,16	8,24	9,50	7,75	47,98	7,41	26,62
	S.aur_HUV05	6	0,12	0,09	0,07	0,06	0,00		90,62	94,86	92,04	94,48	91,46	77,14	76,88	75,98	75,98	77,71	76,62	76,69	92,04	77,14	80,35	76,36	76,24	8,85	8,20	8,20	8,16	8,24	9,50	7,75	47,98	7,41	26,62
	S.equ_C2014	7	0,09	0,10	0,11	0,10	0,10	0,10		91,91	91,65	91,52	91,20	76,49	76,04	75,34	75,59	76,62	75,92	76,17	91,27	76,49	79,38	76,11	75,92	9,06	8,40	8,32	8,40	8,40	9,62	7,75	48,30	7,49	26,28
	S.hom_FDAARGO762	8	0,09	0,07	0,06	0,05	0,05	0,05	0,09		93,96	96,08	93,77	77,33	77,01	76,56	76,36	77,71	77,20	77,07	93,06	77,33	80,28	75,53	75,66	8,89	8,48	8,32	8,28	8,48	9,42	7,99	47,79	7,65	26,58
	S.nep_JS101	9	0,08	0,04	0,07	0,08	0,08	0,08	0,09	0,06		94,35	94,73	77,01	76,49	76,30	75,98	77,71	76,75	76,75	95,89	77,01	80,48	76,36	76,24	9,22	8,64	8,52	8,56	8,60	9,46	7,54	47,73	7,81	26,58
	S.pxyl_14AME19	10	0,10	0,06	0,07	0,05	0,06	0,06	0,09	0,04	0,06		93,71	77,59	77,65	76,81	76,56	78,29	77,20	77,33	93,45	77,59	80,67	76,30	76,17	9,18	8,44	8,36	8,24	8,44	9,66	7,91	47,47	7,49	26,51
	S.sapro_UTI-045	11	0,08	0,06	0,07	0,08	0,09	0,09	0,09	0,07	0,05	0,07		77,07	76,62	76,11	76,17	77,26	76,75	76,88	95,31	77,07	80,73	75,72	75,59	8,85	8,32	8,08	8,16	8,28	9,66	7,54	47,98	7,85	26,43
*hsd*RSMS$_{CHRM}$	S. aur_45394F	12	0,29	0,27	0,28	0,28	0,27	0,27	0,28	0,27	0,27	0,27	0,27		94,09	91,78	92,16	91,97	91,78	92,42	76,75	100,00	88,76	78,29	78,16	8,48	8,40	8,48	8,52	8,48	9,62	7,42	48,11	7,81	26,58
	S. aur_ER02693.3	13	0,29	0,28	0,29	0,29	0,28	0,28	0,29	0,27	0,28	0,27	0,28	0,06		91,78	91,97	92,10	91,27	91,78	76,88	94,09	91,07	77,97	77,46	8,89	8,52	8,60	8,64	8,52	9,85	7,75	47,73	7,81	26,62
	S. cap_SY333	14	0,29	0,28	0,29	0,29	0,29	0,29	0,30	0,28	0,28	0,28	0,29	0,09	0,09		92,36	91,27	92,74	93,96	75,98	91,78	88,76	77,97	77,39	8,65	8,40	8,52	8,44	8,68	9,70	7,50	48,23	8,08	26,36
	S. carn_FDAARGOS_1147	15	0,30	0,28	0,30	0,29	0,29	0,29	0,30	0,28	0,29	0,28	0,29	0,08	0,08	0,08		91,46	92,10	93,13	75,66	92,16	87,73	78,10	77,65	8,77	8,64	8,72	8,60	8,68	9,85	7,59	48,23	7,96	26,39
	S. cond_FDAARGOS1205	16	0,27	0,27	0,28	0,28	0,26	0,26	0,28	0,26	0,26	0,26	0,27	0,08	0,08	0,09	0,09		91,39	91,39	77,14	91,97	89,08	77,65	77,26	8,89	8,92	8,96	8,84	8,92	9,93	7,67	48,30	8,08	26,81
	S. epid_RP62A	17	0,29	0,28	0,29	0,29	0,28	0,28	0,29	0,27	0,28	0,27	0,28	0,09	0,09	0,08	0,08	0,09		94,03	76,24	91,78	87,86	77,65	77,46	8,61	8,52	8,64	8,44	8,68	9,66	7,38	49,31	7,96	26,43
	S. epid_FDAARGOS_161	18	0,28	0,28	0,29	0,28	0,28	0,28	0,29	0,27	0,28	0,27	0,28	0,08	0,09	0,06	0,07	0,09	0,06		76,62	92,42	87,86	78,29	78,03	8,89	8,52	8,68	8,44	8,80	9,38	7,38	48,55	7,92	26,09
	S. equ_FDAARGOS_1149	19	0,08	0,05	0,07	0,08	0,08	0,08	0,09	0,07	0,04	0,07	0,05	0,28	0,28	0,29	0,29	0,27	0,29	0,28		76,75	80,80	76,94	76,81	9,18	8,40	8,36	8,44	8,52	9,54	7,59	47,98	7,61	26,62
	S. hom_TFGsh1	20	0,29	0,27	0,28	0,28	0,27	0,27	0,28	0,27	0,27	0,27	0,27	0,00	0,06	0,09	0,08	0,08	0,09	0,08	0,28		88,76	78,29	78,16	8,48	8,40	8,48	8,52	8,48	9,62	7,42	48,11	7,81	26,58
	S. sapro UTI-042y	21	0,24	0,23	0,23	0,24	0,23	0,23	0,24	0,23	0,23	0,22	0,22	0,12	0,10	0,12	0,13	0,12	0,13	0,13	0,22	0,12		77,26	77,20	9,10	8,48	8,52	8,44	8,44	9,89	7,99	48,99	7,65	26,43
	S. schl_OT1-1	22	0,29	0,28	0,28	0,28	0,28	0,28	0,29	0,30	0,28	0,28	0,29	0,26	0,26	0,26	0,26	0,27	0,27	0,26	0,28	0,26	0,27		97,62	9,02	8,24	8,40	8,40	8,56	9,81	7,59	48,04	8,20	27,00
	M. fleu_FDAARGOS_682	23	0,29	0,28	0,28	0,29	0,29	0,29	0,29	0,29	0,29	0,29	0,30	0,26	0,27	0,27	0,27	0,27	0,27	0,26	0,28	0,26	0,27	0,02		8,98	8,08	8,36	8,32	8,56	9,70	7,46	47,66	8,32	26,96
*hsd*MSR$_{CHRM}$	S.xyl_2	24	1,27	1,28	1,38	1,37	1,36	1,36	1,31	1,35	1,27	1,28	1,36	1,47	1,35	1,42	1,38	1,35	1,43	1,35	1,28	1,47	1,30	1,32	1,33		38,86	38,99	39,17	38,99	12,04	33,86	7,97	33,31	17,97
	S.xyl_TMW 2.1693	25	1,39	1,45	1,46	1,46	1,51	1,51	1,45	1,43	1,38	1,44	1,47	1,45	1,42	1,45	1,38	1,31	1,42	1,42	1,45	1,45	1,43	1,50	1,55	0,94		91,49	88,45	92,08	12,98	33,66	7,86	49,94	19,11
	S.xyl_HKUOPL8	26	1,38	1,46	1,46	1,45	1,51	1,51	1,47	1,47	1,42	1,46	1,55	1,43	1,39	1,42	1,36	1,30	1,38	1,37	1,46	1,43	1,42	1,45	1,46	0,94	0,09		87,92	94,39	13,15	32,99	7,78	51,11	18,93
	S.xyl_S170	27	1,39	1,46	1,50	1,49	1,52	1,52	1,45	1,49	1,40	1,50	1,52	1,42	1,38	1,44	1,39	1,33	1,44	1,44	1,44	1,42	1,44	1,45	1,47	0,93	0,13	0,13		86,67	13,02	33,72	7,82	50,00	19,33
	S.xyl_SMQ121	28	1,36	1,43	1,43	1,40	1,50	1,50	1,45	1,43	1,39	1,44	1,49	1,43	1,42	1,37	1,37	1,31	1,37	1,34	1,42	1,43	1,44	1,40	1,40	0,94	0,08	0,06	0,15		13,20	33,54	7,66	51,17	19,29
Ref-genes	M.EcoKI_typeIA	29	1,25	1,21	1,24	1,24	1,23	1,23	1,21	1,25	1,24	1,20	1,20	1,21	1,16	1,19	1,16	1,14	1,20	1,26	1,22	1,21	1,15	1,17	1,19	1,26	1,24	1,22	1,24	1,21		14,72	9,47	14,43	19,97
	M.EcoAI_typeIB	30	1,66	1,69	1,78	1,64	1,67	1,67	1,67	1,58	1,76	1,61	1,76	1,82	1,67	1,78	1,74	1,71	1,84	1,84	1,74	1,82	1,58	1,74	1,80	1,09	1,16	1,20	1,15	1,16	0,96		8,52	34,86	17,21
	M.EcoR124I_typeIC	31	0,85	0,83	0,84	0,83	0,84	0,84	0,83	0,85	0,85	0,86	0,84	0,84	0,85	0,83	0,83	0,83	0,80	0,82	0,84	0,84	0,81	0,84	0,85	1,62	1,63	1,66	1,65	1,71	1,27	1,44		9,01	26,29
	M.StySBLI_typeID	32	1,68	1,89	1,95	2,02	2,02	2,02	1,97	1,89	1,81	1,97	1,80	1,81	1,81	1,70	1,74	1,70	1,74	1,76	1,91	1,81	1,89	1,65	1,61	1,23	0,73	0,70	0,73	0,69	1,11	1,14	1,41		18,92
	M.KpnBI_typeIE	33	0,99	0,98	0,94	0,95	0,95	0,95	0,97	0,95	0,95	0,96	0,96	0,95	0,95	0,97	0,97	0,94	0,96	0,99	0,95	0,95	0,96	0,93	0,93	1,43	1,35	1,37	1,32	1,33	1,30	1,50	0,97	1,53	

Type I restriction modification subunit *hsd*R

Group	Strain		1	2	3	4	5	6	7	8	9	10	11	12	13	14	15	16	17	18	19	20	21	22	23	24	25	26	27	28	29 typeIA	30 typeIB	31 typeIC	32 typeID	33 typeIE
*hsd*RSMS$_{PL}$	S. xyl_TMW 2.1023	1		96,56	95,84	95,41	95,41	95,41	94,94	95,84	96,27	95,98	95,73	69,22	68,90	68,97	69,04	69,04	68,97	69,00	86,54	69,22	68,54	67,32	67,32	26,09	28,80	28,94	29,03	28,65	23,73	17,80	39,58	25,54	32,12
	S. xyl_TMW 2.1324	2	0,04		97,52	96,95	96,70	96,70	96,13	97,24	96,84	97,45	97,95	69,61	69,15	69,00	69,25	69,36	69,11	69,00	86,94	69,61	68,75	67,43	67,54	26,22	28,48	28,60	28,80	28,31	23,54	17,60	39,77	25,46	32,27
	S.aur_SA01	3	0,04	0,03		98,64	97,06	97,06	96,34	97,70	97,42	97,74	97,17	69,18	68,86	68,97	69,04	69,07	69,00	69,04	86,98	69,18	68,39	67,39	67,39	26,20	28,51	28,62	28,88	28,34	23,41	17,60	40,06	25,63	32,42
	S.aur_55-100-016	4	0,05	0,03	0,01		96,66	96,66	96,05	97,34	96,95	97,52	96,95	69,43	69,04	69,25	69,15	69,18	69,18	69,22	87,05	69,43	68,50	67,39	67,43	26,25	28,62	28,74	28,97	28,42	23,57	17,57	40,03	25,74	32,36
	S.aur_UP966	5	0,05	0,03	0,03	0,03		99,93	95,77	97,92	97,13	97,31	96,77	69,25	68,97	69,15	69,18	69,15	69,07	69,15	86,87	69,25	68,61	67,61	67,57	26,09	28,71	28,85	28,94	28,31	23,49	17,60	39,68	25,51	32,18
	S.aur_HUV05	6	0,05	0,03	0,03	0,03	7,18E-4		95,77	97,92	97,13	97,31	96,77	69,22	68,93	69,11	69,15	69,11	69,04	69,11	86,87	69,22	68,57	67,57	67,54	26,06	28,74	28,88	28,97	28,34	23,49	17,60	39,71	25,51	32,15
	S.equ_C2014	7	0,05	0,04	0,04	0,04	0,04	0,04		96,16	95,84	96,52	96,63	70,08	69,61	69,72	70,08	70,11	69,68	69,57	87,09	70,08	69,50	67,50	67,46	26,14	28,65	28,76	28,96	28,39	23,68	17,51	39,74	25,42	32,23
	S.hom_FDAARGO762	8	0,04	0,03	0,02	0,03	0,02	0,02	0,04		97,42	97,67	97,20	69,36	69,07	69,18	69,25	69,29	69,11	69,18	87,19	69,36	68,72	67,86	67,93	26,20	28,80	28,91	29,00	28,42	23,73	17,78	39,71	25,46	32,33
	S.nep_JS101	9	0,04	0,03	0,03	0,03	0,03	0,03	0,04	0,03		97,52	96,99	69,68	69,29	69,32	69,32	69,50	69,25	69,36	86,87	69,68	68,90	67,54	67,54	26,17	28,91	29,05	29,17	28,48	23,65	17,89	39,80	25,29	32,24
	S.pxyl_14AME19	10	0,04	0,03	0,02	0,03	0,03	0,03	0,03	0,02	0,03		97,45	69,47	69,29	69,40	69,36	69,32	69,25	69,36	86,90	69,47	68,90	67,79	67,71	26,25	28,62	28,77	28,83	28,22	23,43	17,60	39,84	25,54	32,12
	S.sapro_UTI-045	11	0,04	0,02	0,03	0,03	0,03	0,03	0,03	0,03	0,03	0,03		69,72	69,47	69,47	69,50	69,61	69,43	69,40	87,15	69,72	69,00	67,82	67,89	26,31	28,74	28,85	29,03	28,48	23,38	17,69	39,84	25,48	32,33
*hsd*RSMS$_{CHRM}$	S. aur_45394F	12	0,39	0,38	0,39	0,39	0,39	0,39	0,38	0,39	0,38	0,39	0,38		94,81	93,56	96,13	97,64	93,41	92,77	68,14	100,00	95,06	71,74	71,56	25,47	28,18	28,29	28,89	28,69	23,33	16,54	39,42	24,91	32,49
	S. aur_ER02693.3	13	0,40	0,39	0,40	0,39	0,39	0,40	0,38	0,39	0,39	0,39	0,39	0,05		93,77	94,56	94,81	93,48	93,70	67,89	94,81	96,85	71,49	71,60	25,90	28,18	28,32	28,87	28,47	23,19	16,51	39,58	25,02	32,18
	S. cap_SY333	14	0,39	0,39	0,39	0,39	0,39	0,39	0,38	0,39	0,39	0,39	0,39	0,07	0,07		93,16	93,23	97,99	96,92	67,86	93,56	92,62	71,35	71,53	25,80	28,58	28,69	29,21	28,89	23,60	16,45	39,39	25,11	32,33
	S. carn_FDAARGOS_1147	15	0,39	0,39	0,39	0,39	0,39	0,39	0,38	0,39	0,39	0,39	0,39	0,04	0,06	0,07		97,67	93,05	92,59	67,97	96,13	95,06	71,24	71,03	25,63	28,18	28,35	28,89	28,78	23,41	16,66	39,52	25,13	32,12
	S. cond_FDAARGOS1205	16	0,39	0,39	0,39	0,39	0,39	0,39	0,38	0,39	0,39	0,39	0,38	0,02	0,05	0,07	0,02		92,95	92,80	68,04	97,64	95,17	71,56	71,31	25,50	28,18	28,29	28,78	28,67	23,35	16,45	39,55	25,11	32,33
	S. epid_RP62A	17	0,39	0,39	0,39	0,39	0,39	0,39	0,38	0,39	0,39	0,39	0,39	0,07	0,07	0,02	0,07	0,07		96,92	68,00	93,41	92,41	71,03	71,13	25,85	28,52	28,64	29,24	28,78	23,60	16,60	39,70	25,30	32,33
	S. epid_FDAARGOS_161	18	0,39	0,39	0,39	0,39	0,39	0,39	0,38	0,39	0,39	0,39	0,39	0,06	0,07	0,03	0,08	0,08	0,03		68,04	92,77	92,70	71,13	71,13	25,58	28,29	28,35	28,98	28,87	23,63	16,48	39,45	25,08	32,12
	S. equ_FDAARGOS_1149	19	0,15	0,14	0,14	0,14	0,14	0,14	0,14	0,14	0,14	0,14	0,14	0,41	0,41	0,41	0,41	0,41	0,41	0,41		68,14	67,89	66,14	65,93	25,98	27,97	28,08	28,28	28,08	23,35	17,42	39,77	25,79	32,45
	S. hom_TFGsh1	20	0,39	0,38	0,39	0,39	0,39	0,39	0,38	0,39	0,38	0,39	0,38	0,00	0,05	0,07	0,04	0,02	0,07	0,08	0,41		95,06	71,74	71,56	25,47	28,18	28,29	28,89	28,69	23,33	16,54	39,42	24,91	32,49
	S. sapro UTI-042y	21	0,40	0,40	0,40	0,40	0,40	0,40	0,39	0,40	0,40	0,40	0,39	0,05	0,03	0,08	0,05	0,05	0,08	0,08	0,41	0,05		71,24	70,99	25,61	28,01	28,09	28,64	28,09	23,30	16,33	39,17	24,96	32,36
	S. schl_OT1-1	22	0,42	0,42	0,42	0,42	0,42	0,42	0,42	0,41	0,42	0,41	0,41	0,35	0,35	0,36	0,36	0,35	0,36	0,36	0,44	0,35	0,36		97,57	26,42	27,66	27,78	28,38	27,98	23,95	17,75	39,71	25,81	32,55
	M. fleu_FDAARGOS_682	23	0,42	0,42	0,42	0,42	0,42	0,42	0,42	0,41	0,42	0,41	0,41	0,35	0,35	0,35	0,36	0,36	0,36	0,36	0,45	0,35	0,36	0,02		26,37	27,86	27,98	28,69	27,98	23,98	17,95	39,62	25,44	32,15
*hsd*MSR$_{CHRM}$	S.xyl_2	24	1,26	1,25	1,26	1,25	1,26	1,27	1,26	1,26	1,26	1,25	1,25	1,32	1,29	1,29	1,31	1,32	1,29	1,31	1,27	1,32	1,31	1,25	1,25		35,29	35,48	35,68	35,73	25,66	17,39	26,18	30,87	25,76
	S.xyl_2.1693	25	1,13	1,16	1,15	1,15	1,14	1,14	1,14	1,13	1,13	1,15	1,14	1,18	1,18	1,15	1,18	1,18	1,15	1,17	1,19	1,18	1,19	1,22	1,20	0,98		98,56	94,56	92,09	25,38	18,51	27,25	40,00	29,03
	S.xyl_HKUOPL8	26	1,12	1,15	1,15	1,14	1,13	1,13	1,13	1,13	1,12	1,14	1,13	1,17	1,17	1,14	1,17	1,17	1,15	1,17	1,18	1,17	1,18	1,21	1,19	0,97	0,01		95,16	91,64	25,25	18,34	27,11	39,76	29,17
	S.xyl_S170	27	1,12	1,13	1,13	1,12	1,12	1,12	1,12	1,12	1,11	1,13	1,12	1,13	1,13	1,11	1,13	1,14	1,11	1,12	1,17	1,13	1,15	1,17	1,15	0,96	0,06	0,05		91,87	25,59	18,46	27,19	39,91	29,26
	S.xyl_SMQ12	28	1,14	1,17	1,16	1,16	1,17	1,16	1,16	1,16	1,16	1,17	1,16	1,14	1,16	1,13	1,14	1,14	1,14	1,13	1,18	1,14	1,18	1,19	1,19	0,96	0,08	0,09	0,09		25,46	18,66	27,33	41,07	28,94
Ref-genes	R.EcoKI_typeIA	29	1,66	1,68	1,70	1,68	1,69	1,69	1,67	1,66	1,67	1,70	1,71	1,72	1,74	1,68	1,71	1,72	1,68	1,68	1,71	1,72	1,72	1,64	1,64	1,79	1,69	1,70	1,66	1,68		31,02	25,67	28,73	23,89
	R.EcoAI_typeIB	30	1,78	1,82	1,82	1,83	1,82	1,82	1,84	1,79	1,76	1,82	1,80	2,08	2,09	2,11	2,05	2,11	2,07	2,10	1,85	2,08	2,15	1,81	1,77	1,91	1,83	1,87	1,84	1,80	0,91		18,53	20,31	19,61
	R.EcoR124I_typeIC	31	0,95	0,94	0,93	0,93	0,94	0,94	0,94	0,94	0,94	0,94	0,94	0,95	0,95	0,95	0,95	0,95	0,94	0,95	0,94	0,95	0,96	0,95	0,95	1,48	1,41	1,43	1,42	1,41	1,70	1,77		26,67	34,13
	R.StySBLI_typeID	32	1,41	1,42	1,40	1,39	1,41	1,41	1,42	1,42	1,43	1,41	1,42	1,47	1,46	1,46	1,45	1,46	1,44	1,46	1,39	1,47	1,47	1,39	1,43	1,30	1,01	1,02	1,01	0,97	1,50	1,56	1,54		28,81
	R.KpnBI_typeIE	33	1,17	1,16	1,15	1,16	1,17	1,17	1,16	1,16	1,16	1,17	1,16	1,15	1,17	1,16	1,17	1,16	1,16	1,17	1,15	1,15	1,16	1,15	1,18	1,49	1,32	1,31	1,30	1,33	1,83	1,65	1,18	1,37	

Figure S2: neighbor joining tree of *hsd*R from type I RM systems of different bacterial organisms and strains. The turquoise group represents *hsd*R genes of $hsdRSMS_{PL}$ systems, the green group belongs to *S. xylosus* chromosomal *hsdMSR* systems and the group in rose encompasses *hsd*R genes of $hsdRSMS_{CHRM}$ systems. *HsdR* of *S. equorum* FDAARGOS_1149 is chromosomally encoded but clusters with the plasmid-based group. Reference genes of type I systems (A-E) were included into the Figure. The bar indicates 50% sequence divergence.

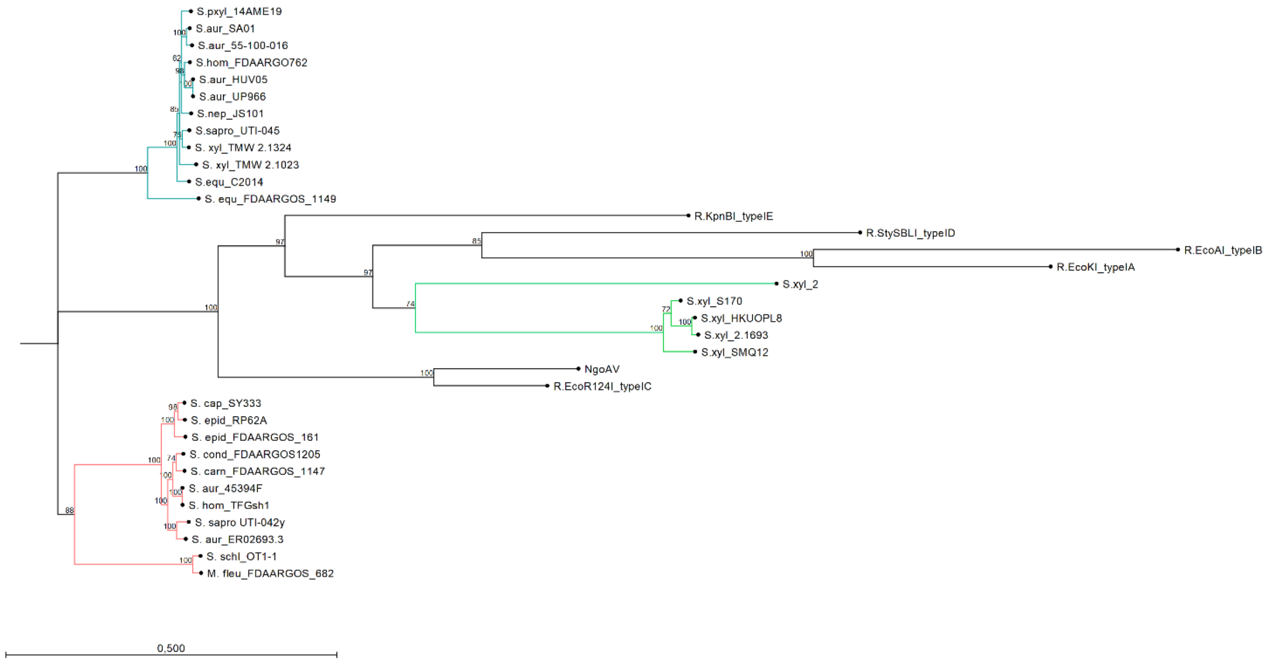

Figure S3: restriction digest (SfaNI) of plasmid (pIMAY*) isolated from *E. coli* strain expressing the *S. xylosus* TMW 2.1324 Type II methyltransferase from different promotors (P_{bla}, P_{N25})

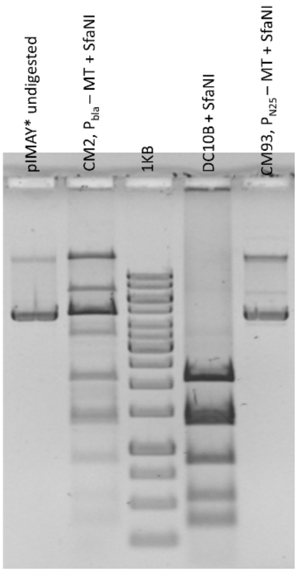

Figure S4: *hsdS_short* alignment

```
                                     20                   40                   60                   80                  100
                                      |                    |                    |                    |                    |
JGY91_13170_2  ATGTTAAAAG ATTATGTTAA TTATCAAAGT GGCACATTAA TACAGCGCTT AGAAGTTGAT GAGAGCGACC TGGGGAATCG TTATATGGTA TATGATCAAA 100
JGY90_14125_2  ATGTTAAAAG ATTATGTTAA TTATCAAAGT GGCACATTAA TACAGCGCTT AGAAGTTGAT GAGAGTGACC TGGGGAATCG TTATATGGTA TATGACCAAA 100
AVJ22_13795_2  ATGTTAAAAG ATTATGTTAA TTATCAAAGT GGCACATTAA TACAACGTTT AGAAGTCGAT GAGAGCGGCA TAGGGAATCA TTATATGGTA TATGATCAAA 100
HSZ48_13155_2  ATGTTAAAAG ATTATGTTAA TTATCAAAGT GGCACATTAA TACAGCGCTT AGAAGTTGAT GAGAGTGACC TGGGGAATCG TTATATGGTA TATGATCAAA 100
BJD96_13980_2  ATGTTAAAAG ATTATGTTAA TTATCAAAGT GGCACATTAA TACAGCGCTT AAAAGTTGAT GAGAGCGACC TGGGGAATCG TTATATGGTA TATGATCAAA 100
E3S94_13565_2  ATGTTAAAAG ATTATGTTAA TTATCAAAGT GGCACATTAA TACAGCGCTT AGAAGTTGAT GATAGCGACC TAGGGAATCG TTATATGATA TATGATCAAA 100
ER12_013985_2  ATGTTAAAAG ATTATGTTAA TTATCAAAGT GGCACATTAA TACAGCGCTT AGAAGTTGAT GATAGCGACC TAGGGAATCG TTATATGATA TATGATCAAA 100
KRH48_13755_2  ATGTTAAAAG ATTATGTTAA TTATCAAAGT GGCACATTAA TACAGCGCTT AGAAGTTGAT GAGAGCGACC TGGGGAATCG TTATATGATA TATGATCAAA 100
HK402_14630_2  ATGTTAAAAG ATTATGTTAA TTATCAAAGT GGCACATTAA TACAGCGCTT AGAAGTTGAT GAGAGTGACC TGGGGAATCG TTATATGGTA TATGATCAAA 100
FOC68_11115_2  ATGTTAAAAG ATTATGTTAA TTATCAAAGT GGCACATTAA TACAGCGCTT AGAAGTTGAT GAGAGCGACC TGGGGCATCG TTATATGATA TATGATCAAA 100
         100%
Conservation
           0%
                                    120                  140                  160                  180                  200
                                      |                    |                    |                    |                    |
JGY91_13170_2  ATATGTTAGA TATAGATTTA GGGTTAACCA TAGATGATAC ATATCAACCT AGAGTCATAA GATTAAAAGA TACATCAAGA GCAACCGTTG TACGTGAAGA 200
JGY90_14125_2  ATATGTTACA TATAGATTTA GGGTTAACCA TAGATGATAC ATATCAACCT AGAGTCATCA AATTAAAAGA TACATCAAGA GCAACCGTTG TACGTAAAGA 200
AVJ22_13795_2  ATATGTTAGA TATAGATTTA GGTTTAACCA TAGATGATAC ATATCAACCT AGAGTCATCA AATTAAAAGA TTCATCAAGA GCAACAGTTA TACGTGAAGA 200
HSZ48_13155_2  ATATGTTAGA TATAGATTTA GGGTTAACCA TAGATGATAC ATATCAACCT AGAGTCATCA AATTAAAAGA TACATCAAGA GCAACCGTTG TACGTAAAGA 200
BJD96_13980_2  ATATGTTAGA TATAGATTTA GGGTTAACCA TAGATGATAC ATATCAACCT AGAGTCATCA AATTAAAAGA TACATCAAGA GCAACCATTG TACGTAAAGA 200
E3S94_13565_2  ATATGCTAGA TATAGATTTA GGGTTAACCA TAGATGATAC ATATCAACCT AGAGTCATCA AATTAAAAGA TACATCAAGA GCAACCGTTG TACGTAAAGA 200
ER12_013985_2  ATATGCTAGA TATAGATTTA GGGTTAACCA TAGATGATAC ATATCAACCT AGAGTCATCA AATTAAAAGA TACATCAAGA GCAACCGTTG TACGTAAAGA 200
KRH48_13755_2  ATATGTTAGA TATAGATTTA GGGTTAACCA TAGATGATAC ATATCAACCT AGAGTCATCA AATTAAAAGA TACATCAAGA GCAACCATTG TACATAAAGA 200
HK402_14630_2  ATATGTTAGA TATAGATTTA GGGTTAACCA TAGATGATAC ATATCAACCT AGAGTCATCA AATTAAAAGA TACATCAAGA GCAACCGTTG TACGTAAAGA 200
FOC68_11115_2  ATATGCTAAA TATAGATTTA GGGTTAACCA TAGATGATAC ATATCAACCT AGAGTCATCA AATTAAAAGA TACATCAAGA GCAACCGTTG TACATAAAGA 200
         100%
Conservation
           0%
                                    220                  240                  260                  280                  300
                                      |                    |                    |                    |                    |
JGY91_13170_2  CGACATTGTG TTTAGTATGA TGACAGGTGA ATGTACTCTA GTGAGTTCGA AACATGATGG TTCAATTTTA CCTTATAACT ATACAAAAAT TGAAGTTACG 300
JGY90_14125_2  TGATATTGTC ATAAGTATGA TGACAGGTGA GTGCACCTTA GTGAGCGCAA GACACGATGG TTCAATTTTA CCTTATAACT ATACAAAAAT TGAAGTTACT 300
AVJ22_13795_2  CGATATTGTG TTTAGTATGA TTACAGGTGA ATGTACTTTA GTGAGTTCGA AACATGATGG CTCAATCTTA CCTTATAATT ATACAAAAAT TGAAGTTACC 300
HSZ48_13155_2  TGATATTGTC ATAAGTATGA TGACAGGTGA GTGCACCTTA GTGAGCGCAA GACACGATGG TTCAATTTTA CCTTATAACT ATACAAAAAT TGAAGTTACT 300
BJD96_13980_2  TGATATTGTC ATAAGTATGA TGACAGGTGA GTGCACCTTA GTGAGTGCAA GACACGATGG TTCAATTTTA CCTTATAACT ATACAAAAAT TGAAGTTACT 300
E3S94_13565_2  TGATATTGTC ATAAGTATGA TGACAGGTGA GTGCACCCTA GTGAGCACAA GACACGATGG TTCAATTTTA CCTTATAACT ATACAAAAAT TGAAGTTACT 300
ER12_013985_2  TGATATTGTC ATAAGTATGA TGACAGGTGA GTGCACCCTA GTGAGCACAA GACACGATGG TTCAATTTTA CCTTATAACT ATACAAAAAT TGAAGTTACT 300
KRH48_13755_2  TGATATTGTT ATAAGTATGA TGACAGGTGA GTGCACCTTA GTGAGCACAA AACACGATGG TTCAATTTTA CCTTATAACT ATACAAAAAT TGAAGTTACT 300
HK402_14630_2  TGATATTGTC CTAAGTATGA TGACAGGTGA GTGCACCTTA GTGAGCTCAA GACACGATGG TTCAATTTTA CCTTATAACT ATACAAAAAT TGAAGTTACT 300
FOC68_11115_2  TGATATTGTC ATAAGTATGA TGACAGGTGA GTGCACCTTA GTGAGCACAA GACACGATGG TTCAATTTTA CCTTATAACT ATACAAAAAT TGAAGTAACT 300
         100%
Conservation
           0%
                                    320                  340                  360                  380                  400
                                      |                    |                    |                    |                    |
JGY91_13170_2  TCTGAATTAT TGGAACCTAC TTTTTTAGTC TATTGGTTTC AACTAGCACC AGAGGTACAA TCACAATATA AGCAATATAT GCAGGGTGGC TCAACAATTA 400
JGY90_14125_2  TCAGACTTAT TGGAACCTGC TTTTTTAGTC TATTGGTTTC AACTAGCACC AGAGGTACAA TCACAATATA AGCAATATAT GCAAGGTGGC TCAACAATAA 400
AVJ22_13795_2  TCTGAATTGT TGGAACCTAC TTTTTTAGTC TATTGGTTTC AATTAGCACC AGAAGTACAA TCACAATATA AGCAATATAT GCAGGGCGGC TCAACAATTA 400
HSZ48_13155_2  TCAGACTTAT TGGAACCTGC TTTTTTAGTC TATTGGTTTC AACTAGCACC AGAGGTACAA TCACAATATA AGCAATATAT GCAAGGTGGC TCAACAATAA 400
BJD96_13980_2  TCAGACTTAT TGGAACCTGC TTTTTTAGTC TATTGGTTTC AACTAGCACC AGAGGTACAA TCACAATATA AGCAATATAT GCAGGGTGGC TCAACAATAA 400
E3S94_13565_2  TCAGACTTAT TGGAACCTGC TTTTTTAGTC TATTGGTTTC AACTAGCACC AGAGGTACAA GTACAATATA AGCAATATAT GCAGGGTGGC TCAACAATAA 400
ER12_013985_2  TCAGACTTAT TGGAACCTGC TTTTTTAGTC TATTGGTTTC AACTAGCACC AGAGGTACAA GTACAATATA AGCAATATAT GCAGGGTGGC TCAACAATAA 400
KRH48_13755_2  TCAGACTTAT TGGAACCTGC TTTTTTAGTC TATTGGTTTC AACTAGCACC AGAGGTACAA TCACAATATA AGCAATATAT GCAGGGTGGC TCAACAATAA 400
HK402_14630_2  TCAGACTTAT TGGAACCTGC TTTTTTAGTC TATTGGTTTC AACTAGCACC AGAGGTACAA TCACAATATA AGCAATATAT GCAAGGTGGC TCAACAATAA 400
FOC68_11115_2  TCAGACTTAT TGGAACCTGC TTTTTTAGTC TATTGGTTTC AACTAGCACC AGAGGTACAT TCACAATATA AGCAATATAT GCAGGGTGGC TCAACAATAA 400
         100%
Conservation
           0%
                                    420                  440                  460                  480                  500
                                      |                    |                    |                    |                    |
JGY91_13170_2  AAAAATTAAC CCACCAACAG TTAAAATCAT TACACATAAC TTTACCTACT TTAGAGAAAC AAAGATTGAT TGGATATATC GGGGTTAAAG AAAAACGATT 500
JGY90_14125_2  AAAAACTAAC ACATCAACAG TTAAAATCAC TCTATATTAC CTTACCTTCA ATTGAACGAC AACAGTTGAT TGGGCAAATT GGTATTAAAG AAAAACAATT 500
AVJ22_13795_2  AAAAATTAAC CCACCAACAG TTAAAATCAT TACACATAAC TTTACCTACT TTAGAGAAAC AAAGATTGAT TGGATATATC GGGGTTAAAG AAAAACGATT 500
HSZ48_13155_2  AAAAACTAAC GCATCAACAG TTAAAATCAC TCTACATTAC CTTACCTTCA ATTGAACGAC AACGGTTGAT TGGACAAATT GGTATTAAAG AAAAACAATT 500
BJD96_13980_2  AAAAACTAAC ACATCAACAG TTAAAATCAC TTTATATTAC TTTACCTTCC ATTGAACGAC AACGGTTGAT TGGGCAAATT GGTATTAAAG AAAAACAATT 500
E3S94_13565_2  AAAAATTAAC ACATCAACAG TTAAAATCAC TCTATATTAC CTTACCTTCA ATTAAACGAC AACGGTTGAT TGGACAAATT GGTATTAAAG AAAAACAATT 500
ER12_013985_2  AAAAATTAAC ACATCAACAG TTAAAATCAC TCTATATTAC CTTACCTTCA ATTAAACGAC AACGGTTGAT TGGACAAATT GGTATTAAAG AAAAACAATT 500
KRH48_13755_2  AAAAACTAAC ACATCAACAG TTAAAATCAC TCTATATTAC CTTACCTTCA ATTGAACGAC AACGGTTGAT TGGACAAATT GGTATTAAAG AAAAACAATT 500
HK402_14630_2  AAAAACTAAC ACATCAACAG TTAAAATCAC TCTATATTAC CTTACCTTCA ATTGAACGAC AACGGTTGAT TGGGCAAATT GGTATTAAAG AAAAACAATT 500
FOC68_11115_2  AGAAACTAAC ACATCAACAG TTAAAATCAC TCTATATTAC CTTACCTTCA ATTGAACGAC AACGGCTGAT TGGGCAAATT GGTATTAAAG AAAAACAATT 500
         100%
Conservation
           0%
                                    520                  540                  560
                                      |                    |                    |
JGY91_13170_2  GAATATCTTA AAACAAAAAC AAAGCCGATT AAAAAAACAA TTTTTAACAA ACACTTTATT CAAGGAGGAC AATTAA 576
JGY90_14125_2  AAACATATTA AAACAAAGAC AAAGTCGGTT AAAAAAGCAA TTCTTATCCA ACACTTTATT CAAGGAGGAC AATTAA 576
AVJ22_13795_2  GAATATATTA AAACAAAAAC AAAGCCGATT AAAAAAACAA TTTTTAACAA ACACTTTATT CAAGGAGGAC AAATAA 576
HSZ48_13155_2  AAACGTATTA AAACAAAGAC AAAGTCGATT AAAAAAGCAA TTCTTATCCA ACACTTTATT CAAGGAGGAC AATTAA 576
BJD96_13980_2  AAACGTATTA AAACAAAGAC AAAGTCGATT AAAAAAGCAA TTCTTATCCA ACACTTTATT CAAGGAGGAC AATTAA 576
E3S94_13565_2  AAACATATTA AAACAAAGAC AAAGTCGATT AAAAAAGCAA TTTTTATCCA ACACTTTATT CAAGGAGGAC AATTAA 576
ER12_013985_2  AAACATATTA AAACAAAGAC AAAGTCGATT AAAAAAGCAA TTTTTATCCA ACACTTTATT CAAGGAGGAC AATTAA 576
KRH48_13755_2  AAACGTATTA AAACAAAGAC AAAGTCGATT AAAAAAGCAA TTCTTATCCA ACACTTTATT CAAGGAGGAC AATTAA 576
HK402_14630_2  AAACGTATTA AAACAAAGGC AAAGTCGATT AAAAAAGCAA TTCTTATCTA ACACTTTATT CAAGGAGGAC AATTAA 576
FOC68_11115_2  AAACGTATTA AAACAAAGAC AAAGTCGATT AAAAAAGCAA TTCTTATCTA ACACTTTATT CAAGGAGGAC AATTAA 576
         100%
Conservation
           0%
```

Figure S5: HsdM (HsdRSMS systems) alignment

```
                                        20                    40                    60                    80
                                         |                     |                     |                     |
S. xyl_TMW 2.1324  MSITEKQRQQ QAELQKNLWS IADDLRGNMD ANEFKNYILG MIFYRFLSEK IEEQAQILLA EDNIDYETAM ADEDYRPVLE QEFISRIGYV 90
S. xyl_TMW 2.1023  MSITEKQRQQ QAELQKNLWS IADDLRGNMD ANEFKNYILG MIFYRFLSEK IEEQAQILLA EDSIDYETAM ADEDYRPVLE QEFISRIGYV 90
S.equ_C2014        MSITEKQRQQ QAELQKNLWS IADDLRGNMD ANEFKNYILG MIFYRFLSEK IEEQAQILLA EDNIDYETAM ADEDYRPVLE QEFISRIGYV 90
S.sapro_UTI-045    MSITEKQRQQ QAELQKNLWS IADDLRGNMD ANEFKNYILG MIFYRFLSEK IEEQAQILLA EDNIDYETAM ADEDYRPVLE QEFISRIGYV 90
S.nep_JS101        MSITEKQRQQ QAELQKNLWS IADDLRGNMD ANEFKNYILG MIFYRFLSEK IEEQAQILLA EDNIDYETAM ADEDYRPVLE QEFISRIGYV 90
S.aur_UP966        MSITEKQRQQ QAELQKNLWS IADDLRGNMD ANEFKNYILG MIFYRFLSEK IEEQAQILLA EDNIDYETAM ADEDYRPVLE QEFISRIGYV 90
S.aur_HUV05        MSITEKQRQQ QAELQKNLWS IADDLRGNMD ANEFKNYILG MIFYRFLSEK IEEQAQILLA EDNIDYETAM ADEDYRPVLE QEFISRIGYV 90
S.aur_55-100-016   MSITEKQRQQ QAELQKNLWS IADDLRGNMD ANEFKNYILG MIFYRFLSEK IEEQAQILLA EDNIDYETAM ADEDYRPVLE QEFISRIGYV 90
S.aur_SA01         MSITEKQRQQ QAELQKNLWS IADDLRGNMD ANEFKNYILG MIFYRFLSEK IEEQAQILLA EDNIDYETAM ADEDYRPVLE QEFISRIGYV 90
S.hom_FDAARGO762   MSITEKQRQQ QAELQKNLWS IADDLRGNMD ANEFKNYILG MIFYRFLSEK IEEQAQILLA EDNIDYETAM ADEDYRPVLE QEFISRIGYV 90
S.pxyl_14AME19     MSITEKQRQQ QAELQKNLWS IADDLRGNMD ANEFKNYILG MIFYRFLSEK IEEQAQILLA EDNIDYETAM ADEDYRPVLE QEFISRIGYV 90
Conservation (100% / 0%)

                            100                   120                   140                   160                   180
                              |                     |                     |                     |                     |
S. xyl_TMW 2.1324  IEPQYLFSHL VKKIEKQVFE MEDLSNAIKN IENSTRGHDS EDDFIHLFDD LDLNSSRLGN SNAARTKLIS KVMMKISTLP FVHSDMEIDM 180
S. xyl_TMW 2.1023  IDPQYLFSHL VKKIERQDFE MEDLSNAIKN IENSTRGHDS EDDFIHLFDD LDLVSSRLGN SNAARTKLIS KVIMKISTLP FVHSDMEIDM 180
S.equ_C2014        IDPQYLFSHL VKKIEKQSFE MEDLSNAIKN IENSTRGHDS EDDFIHLFDD LDLVSSRLGN SNAARTKLIS KVIMKISTLP FVHSDMEIDM 180
S.sapro_UTI-045    IEPQYLFSHL VKKIEKQVFE MEDLSNAIKN IENSTRGHDS EDDFIHLFDD LDLNSSRLGN SNAARTKLIS KVMMKISTLP FVHSDMEIDM 180
S.nep_JS101        IEPQYLFSHL VKKIEKQVFE MEDLSNAIKN IENSTRGHDS EDDFIHLFDD LDLNSSRLGN SNAARTKLIS KVMMKISTLP FVHSDMEIDM 180
S.aur_UP966        IEPQYLFGHL VKKIEKQAFE MEDLSNAIKN IENSTRGHDS EDDFIHLFDD LDLNSSRLGN SNAARTKLIS KVMMKISTLP FVHSDMEIDM 180
S.aur_HUV05        IEPQYLFGHL VKKIEKQAFE MEDLSNAIKN IENSTRGHDS EDDFIHLFDD LDLNSSRLGN SNAARTKLIS KVMMKISTLP FVHSDMEIDM 180
S.aur_55-100-016   IEPQYLFGHL VKKIEKQAFE MEDLSNAIKN IENSTRGHDS EDDFIHLFDD LDLNSSRLGN SNAARTKLIS KVMMKISTLP FVHSDMEIDM 180
S.aur_SA01         IEPQYLFGHL VKKIEKQAFE MEDLSNAIKN IENSTRGHDS EDDFIHLFDD LDLNSSRLGN SNAARTKLIS KVMMKISTLP FVHSDMEIDM 180
S.hom_FDAARGO762   IEPQYLFGHL VKKIEKQAFE MEDLSNAIKN IENSTRGHDS EDDFIHLFDD LDLNSSRLGN SNAARTKLIS KVMMKISTLP FVHSDMEIDM 180
S.pxyl_14AME19     IEPQYLFSHL VKKIEKQVFE MEDLSNAIKN IENSTRGHDS EDDFIHLFDD LDLNSSRLGN SNAARTKLIS KVMMKISTLP FVHSDMEIDM 180
Conservation (100% / 0%)

                                       200                   220                   240                   260
                                         |                     |                     |                     |
S. xyl_TMW 2.1324  LGDAYEYLIG QFAASSGKKA GEFYTPQQVS TILAKIVTAN KKDLKSVYDP TCGSGSLLLR VGREADVRQY YGQEYNSTTY NLARMNMLLH 270
S. xyl_TMW 2.1023  LGDAYEYLIG QFAASSGKKA GEFYTPQQVS TILAKIVTAN KKDIKSVYDP TCGSGSLLLR VGREANVRQY YGQEYNSTTY NLARMNMLLH 270
S.equ_C2014        LGDAYEYLIG QFAASSGKKA GEFYTPQQVS TILAKIVTAN KKDIKSVYDP TCGSGSLLLR VGREADVRQY YGQEFNSTTY NLARMNMLLH 270
S.sapro_UTI-045    LGDAYEYLIG QFAASSGKKA GEFYTPQQVS TILAKIVTAN KKDIKSVYDP TCGSGSLLLR VGREANVRQY YGQEYNSTTY NLARMNMLLH 270
S.nep_JS101        LGDAYEYLIG QFAASSGKKA GEFYTPQQVS TILAKIVTAN KKDLKSVYDP TCGSGSLLLR VGREADVRQY YGQEYNSTTY NLARMNMLLH 270
S.aur_UP966        LGDAYEYLIG QFAASSGKKA GEFYTPQQVS TILAKIVTAN KKDLKSVYDP TCGSGSLLLR VGREANVRQY YGQEYNSTTY NLARMNMLLH 270
S.aur_HUV05        LGDAYEYLIG QFAASSGKKA GEFYTPQQVS TILAKIVTAN KKDLKSVYDP TCGSGSLLLR VGREANVRQY YGQEYNSTTY NLARMNMLLH 270
S.aur_55-100-016   LGDAYEYLIG QFAASSGKKA GEFYTPQQVS TILAKIVTAN KKDIKSVYDP TCGSGSLLLR VGREANVRQY YGQEYNSTTY NLARMNMLLH 270
S.aur_SA01         LGDAYEYLIG QFAASSGKKA GEFYTPQQVS TILAKIVTAN KKDIKSVYDP TCGSGSLLLR VGREANVRQY YGQEYNSTTY NLARMNMLLH 270
S.hom_FDAARGO762   LGDAYEYLIG QFAASSGKKA GEFYTPQQVS TILAKIVTVN KKDIKSVYDP TCGSGSLLLR VGREANVRQY YGQEYNSTTY NLARMNMLLH 270
S.pxyl_14AME19     LGDAYEYLIG QFAASSGKKA GEFYTPQQVS TILAKIVTAN KKDLKSVYDP TCGSGSLLLR VGREANVRQY YGQEYNSTTY NLARMNMLLH 270
Conservation (100% / 0%)

                            280                   300                   320                   340                   360
                              |                     |                     |                     |                     |
S. xyl_TMW 2.1324  DVNYANFKIE NGDTIEDPAI SDERFEAVVA NPPYSAKWSS DPQFLEDPRF SNYGKLAPKS KADFAFIQHM IYHLDDNGTM AVVLPHGVLF 360
S. xyl_TMW 2.1023  DVNYANFKIE NGDTIEDPAI SDERFEAVVA NPPYSAKWSS DPQFLEDPRF SNYGKLAPKS KADFAFIQHM IYHLDDNGTM AVVLPHGVLF 360
S.equ_C2014        DVNYANFKIE NGDTIEDPAI SDERFEAVVA NPPYSAKWSS DPQFLEDPRF SNYGKLAPKS KADFAFIQHM IYHLDDNGTM AVVLPHGVLF 360
S.sapro_UTI-045    DVNYANFKIE NGDTIEDPAI SDERFEAVVA NPPYSAKWSS DPQFLEDPRF SNYGKLAPKS KADFAFIQHM IYHLDDNGTM AVVLPHGVLF 360
S.nep_JS101        DVNYANFKIE NGDTIEDPAI SDERFEAVVA NPPYSAKWSS DPQFLEDPRF SNYGKLAPKS KADFAFIQHM IYHLDDNGTM AVVLPHGVLF 360
S.aur_UP966        DVNYANFKIE NGDTIEDPAI SDERFEAVVA NPPYSAKWSS DPQFLEDPRF SNYGKLAPKS KADFAFIQHM IYHLDDNGTM AVVLPHGVLF 360
S.aur_HUV05        DVNYANFKIE NGDTIEDPAI SDERFEAVVA NPPYSAKWSS DPQFLEDPRF SNYGKLAPKS KADFAFIQHM IYHLDDNGTM AVVLPHGVLF 360
S.aur_55-100-016   DINYANFKIE NGDTIEDPAI SDERFEAVVA NPPYSAKWSS DPQFLEDPRF SNYGKLAPKS KADFAFIQHM IYHLDDNGTM AVVLPHGVLF 360
S.aur_SA01         DVNYANFKIE NGDTIEDPAI SDERFEAVVA NPPYSAKWSS DPQFLEDPRF SNYGKLAPKS KADFAFIQHM IYHLDDNGTM AVVLPHGVLF 360
S.hom_FDAARGO762   DVNYANFKIE NGDTIEDPAI SDERFEAVVA NPPYSAKWSS DPQFLEDPRF SNYGKLAPKS KADFAFIQHM IYHLDDNGTM AVVLPHGVLF 360
S.pxyl_14AME19     DVNYANFKIE NGDTIEDPAI SDERFEAVVA NPPYSAKWSS DPQFLEDPRF SNYGKLAPKS KADFAFIQHM IYHLDDNGTM AVVLPHGVLF 360
Conservation (100% / 0%)

                                       380                   400                   420                   440
                                         |                     |                     |                     |
S. xyl_TMW 2.1324  RGAAEGVIRQ YLIKEKNYLD AIIGLPSNLF FGTSIPTSIL VFKKCREADD NVLFIDASQS FEKGKNQNHL TTEDVEKIVE TYKNRETLDK 450
S. xyl_TMW 2.1023  RGAAEGVIRQ YLIKEKNYLD AIIGLPSNLF FGTSIPTSIL VFKKCREADD NVLFIDASQS FEKGKNQNHL TTEDVEKIVE TYKNRETLDK 450
S.equ_C2014        RGAAEGVIRQ YLIKEKNYLD AVIGLPANLF FGTSIPTSIL VFKKCREVDD NVLFIDASQS FEKGKNQNHL TTEDVEKVVE TYKNRETLDK 450
S.sapro_UTI-045    RGAAEGVIRE YLIKEKNYLD AVIGLPSNLF FGTSIPTSIL VFKKCREDDD NVLFIDASQS FEKGKNQNHL TTEDVEKIVE TYKNRETLDK 450
S.nep_JS101        RGAAEGVIRQ YLIKEKNYLD AIIGLPSNLF FGTSIPTSIL VFKKCREEDD NVLFIDASQS FEKGKNQNHL TTEDVEKIVE TYKNRETLDK 450
S.aur_UP966        RGAAEGVIRE YLIKEKNYLD AVIGLPANLF FGTSIPTSIL VFKKCREDDD NVLFIDASQS FEKGKNQNHL TTEDVEKIVE TYKNRETLDK 450
S.aur_HUV05        RGAAEGVIRE YLIKEKNYLD AVIGLPANLF FGTSIPTSIL VFKKCREDDD NVLFIDASQS FEKGKNQNHL TTEDVEKIVE TYKNRETLDK 450
S.aur_55-100-016   RGAAEGVIRE YLIKEKNYLD AVIGLPSNLF FGTSIPTSIL VFKKCREDDD NVLFIDASQS FEKGKNQNHL TSEDVEKIVE TYKNRETLDK 450
S.aur_SA01         RGAAEGVIRQ YLIKEKNYLD AIIGLPSNLF FGTSIPTSIL VFKKCREADD NVLFIDASQS FEKGKNQNHL TTEDVEKIVE TYKNRETLDK 450
S.hom_FDAARGO762   RGAAEGVIRE YLIKEKNYLD AVIGLPANLF FGTSIPTSIL VFKKCREDDD NVLFIDASQS FEKGKNQNHL TTEDVEKIVE TYKNRETLDK 450
S.pxyl_14AME19     RGAAEGVIRE YLIKEKNYLD AVIGLPANLF FGTSIPTSIL VFKKCREDDD NVLFIDASQS FEKGKNQNHL TSEDVEKIVE TYKNRETLDK 450
Conservation (100% / 0%)

                            460                   480                   500
                              |                     |                     |
S. xyl_TMW 2.1324  YSYAASLKEI AENDYNLNIP RYVDTFEEEE PIDLEQVQKD LNKIDDEIVE VEQEINGYLK ELGVLKHD- 518
S. xyl_TMW 2.1023  YSYVASLEEI AENDYNLNIP RYVDTFEEEE PIDLEQVQKD LNQIDDEIVK VEQEINSYLK ELGVLNDD- 518
S.equ_C2014        YSYAASLEEI AENDYNLNIP RYVDTFEEEE PIDLEQVQKD LNQIDDEIVE VEQEINNYLK ELGVLKHD- 518
S.sapro_UTI-045    YSYAASLEEI AENDYNLNIP RYVDTFEEEE PVDLEQVQKD LNQIDDEIIE VEQEINNYLK ELGVLKHD- 518
S.nep_JS101        YSYAASLEEI AENDYNLNIP RYVDTFEEEE PIDLEQVQKD LNQIDDEIVE VEQEINNYLK ELGVLKHD- 518
S.aur_UP966        YSYAASLEEI TENDYNLNIP RYVDTFEEEE QIDLEQVQSD LKTIEAEIKE VEQEIESYLK ELGVVTHD- 518
S.aur_HUV05        YSYAASLEEI TENDYNLNIP RYVDTFEEEE QIDLEQVQSD LKTIEAEIKE VEQEIESYLK ELGVVTHD- 518
S.aur_55-100-016   YSYAASLEEI AENDYNLNIP RYVDRFEEEE PIDLEQVQKD LNQIDDEIVK VEQEIEGYLK ELGVLKHD- 518
S.aur_SA01         YSYAASLEEI AENDYNLNIP RYVDTFEEEE PIDLEQVQKD LNSIDDEIKA VEQEIEGFLK ELGVVTHD- 518
S.hom_FDAARGO762   YSYAASLEEI AENDYNLNIP RYVDTFEEEE PIDLEQVQKD LNQIDDEIIE VEQEINNYLK ELGVLKHD- 518
S.pxyl_14AME19     YSYAASLEEI AENDYNLNIP RYVDTFEEEE PIDLEQVQKD LNQIDDEIVE VEQEINNYLK ELGVLKHD- 518
Conservation (100% / 0%)
```

Figure S6: Sequence and conserved motif analysis of the adenine-N6-methyltransferase of *S. xylosus* TMW 2.1324 revealed its affiliation to the group alpha MTases of the Dam family (Motif order X-I-II-III-TRD-VI-V-VIVII-VIIII). According to InterPro the sequence consists of two methylation domains (MT1, MT2) which are not further specified but likely represent the two active halves, each recognizing one of the two target motifs of the enzyme (GCATC/GATGC). In *E. coli* CM13, the MTase is disrupted by an IS Element, therefore the sequence misses 173 aa in its C-terminal part. This resulted in hemi-methylation of the DNA.

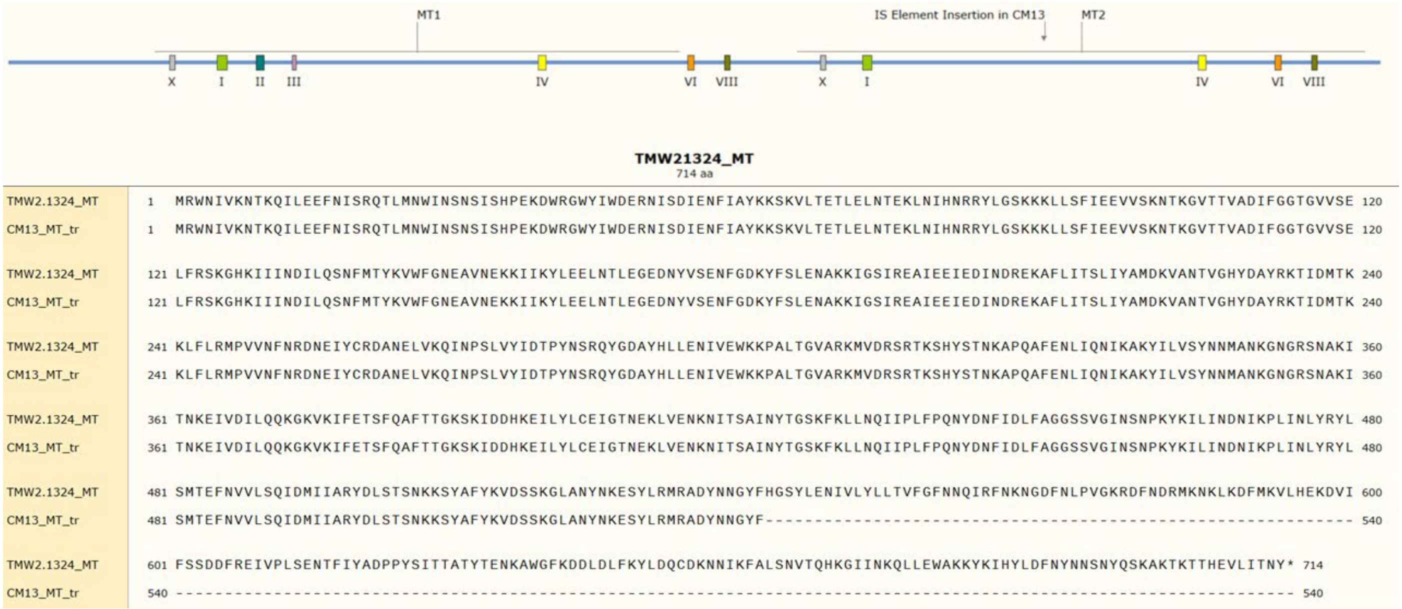

Table S1: Bacterial strains, plasmids and oligonucleotides used in this study. Underlined are overhangs for restriction sites. NCBI accession numbers are provided for whole genome sequenced strains.

Primer	Sequence (5'-3')	Source
vec_pBla_1F	GGATCGGAATTCGAGCTCGGTACTCTACATCTAAACTAAATACTATTGAG	this study
Bla_Mtase_1R	TTCACAATATTCCACCTCATACTCTTCCTTTTTCAATATTATTG	this study
Bla_Mtase_2F	AATATTGAAAAAGGAAGAGTATGAGGTGGAATATTGTG	this study
Mtase_186_2R	CATGCATCTCGAGGCATGCCTGCATTAATAGTTAGTTATTAGTACTTCATG	this study
Pn25_F	CATAAAAAATTTATTTGCTTTCAGGAAAATTTTTCTGTATAATAGATTCATAAATTTGAGAGAGGAGTT	this study
Pn25_R	AACTCCTCTCTCAAATTTATGAATCTATTATACAGAAAAATTTTCCTGAAAGCAAATAAATTTTTTG	this study
SacI_PN25_F	CGAGCTCGCATAAAAAATTTATTTGC	this study
PN25_MT_F	CATAAATTTGAGAGAGGAGTTATGAGGTGGAATATTGTG	this study
RS_MT_R	CCAATGCATTGGTTCTGCAGTTTTAATAGTTAGTTATTAGTACTTCATG	this study
RS_PN25_F	GGGGTACCCCCATAAAAAATTTATTTGC	this study
PN25_hsdSMS_F	GTATAATAGATTCATAAATTTGAGAGAGGAGTTATGTTAAAAGATTATGTTAATTATC	this study
PN25_hsdMS_F	GTATAATAGATTCATAAATTTGAGAGAGGAGTTATGTCTATTACGGAAAAACAAC	this study
RS_hsdS_R	GATCAGCATGCCTACACAAACATCTTCTG	this study
RS_hsdS_tr_R	GATCAGCATGCTTATGCCATTGACTGG	this study
186_1_P1	CTCATTCGAAACCACCCACCG	Cui and Shearwin (2017)
186_1_P2	ACTTAACGGCTGACATGG	Cui and Shearwin (2017)
186_1_P3	ACGAGTATCGAGATGGCA	Cui and Shearwin (2017)
186_1_P4	GATCATCATGTTTATTGCGTGG	Cui and Shearwin (2017)
186_2_P1	TCCGGAATGCCTGCATTG	Cui and Shearwin (2017)
186_2_P2	ACTTAACGGCTGACATGG	Cui and Shearwin (2017)
186_2_P3	ACGAGTATCGAGATGGCA	Cui and Shearwin (2017)
186_2_P4	CCCTGGAGCCAAAATATCC	Cui and Shearwin (2017)
Lambda_P1	GGCATCACGGCAATATAC	Cui and Shearwin (2017)
Lambda_P2	ACTTAACGGCTGACATGG	Cui and Shearwin (2017)
Lambda_P3	GGGAATTAATTCTTGAAGACG	Cui and Shearwin (2017)
Lambda_P4	TCTGGTCTGGTAGCAATG	Cui and Shearwin (2017)
hsdS_s_F_RT	AGATACATCAAGAGCAACCGTTGTACG	this study
hsdS_s_R_RT	GATTGTACCTCTGGTGCTAGTTGAAACC	this study
hsdM_F_RT	GTTGCCAATCCACCTTATAGTGCG	this study
hsdM_R_RT	ACACCTTCAGCTGCGCCAC	this study
hsdS_023_F_RT	GCAAACTCGACCGACAAATCGAATTG	this study
hsdS_023_R_RT	CCCATTCTGGATAATCATTCCCATTCTCATC	this study
hsdS_324_F_RT	GCTCGGAAGAACAGGGATAATGTCACC	this study
hsdS_324_R_RT	GTTCTTCAAACGCAGGCATCATAATAGGC	this study
recA_F	GGCCTTGCGGCACGTATG	this study
recA_R	GCGCCGATACGACGGATG	this study
cysG_F	CGGCGGTGGTGATGTCG	this study
cysG_R	GCGTCATCATCCGTCGCTG	this study
hcaT_F_RT	CGAACCAGCGCCAACCAC	this study
hcaT_R_RT	GGCTCGGTGGCGTTTGTC	this study

strains	Description	Source, Accession
DC10B	*E. coli* DH10B (K12 derivate), Δ*dcm*	Monk et al., (2015)
CM56	*E. coli* DC10B with 2.1023 *hsd*SMS integrated at 186-2 (Promotor: P_{N25})	this study
CM13	*E. coli* DC10B with 2.1324 *hsd*SMS integrated at 186-1 and 2.1324 MT integrated at λ (Promotor: P_{N25})	this study
CM57	*E. coli* DC10B with 2.1023 *hsd*MS integrated at 186-2 (Promotor: P_{N25})	this study
CM19	*E. coli* DC10B with 2.1324 *hsd*MS integrated at 186-1 and 2.1324 MT integrated at λ (Promotor: P_{N25})	this study
CM5	*E. coli* DC10B with 2.1324 *hsd*SMS integrated at 186-1 (Promotor: P_{N25})	this study
CM30	*E. coli* DC10B with 2.1324 *hsd*MS*_tr* integrated at 186-2, 2.1324 MT integrated at λ (Promotor: P_{N25})	this study
CM93	*E. coli* DC10B with 2.1324 MT integrated at λ (Promotor: P_{N25})	this study
CM2	*E. coli* DC10B with 2.1324 MT integrated at 186-1 (Promotor: P_{bla})	this study
DC3.1	*E. coli* resistant to ccdB	Cui and Shearwin (2017)
E811	*E. coli* (P2 lysogen) in which the strong promotor pE is repressed	Cui and Shearwin (2017)
TMW 2.1023	*S. xylosus* isolated from raw fermented sausages	JAEMUG000000000
TMW 2.1324	*S. xylosus* isolated from raw fermented sausages	CP066726-CP066729
TMW 2.1521	*S. xylosus* isolated from raw fermented sausages	JAEMUF000000000
TMW 2.1523	*S. xylosus* isolated from raw fermented sausages	CP066721-CP066725
TMW 2.1602	*S. xylosus* isolated from raw fermented sausages	CP066719-CP066720
TMW 2.1693	*S. xylosus* isolated from bovine mastitis	JAJAGM000000000
TMW 2.1704	*S. xylosus* isolated from bovine mastitis	JAJAGL000000000
TMW 2.1780	*S. xylosus* isolated from raw fermented sausages	JAJAGN000000000
2	*S. xylosus* isolated from Milker's hand, Brazil	CP031275-CP031276
DMSX03	*S. xylosus* isolated from Meju, fermented soybean	CP060271-CP060272
HKUOPL8	*S. xylosus* isolated from animal faecal matter	CP007208-CP007209
S170	*S. xylosus* isolated from leaf vegetable	CP013922
SMQ-121	*S. xylosus* isolated from meat starter culture, Canada	CP008724
C2a	*S. xylosus* isolated from human skin	LN554884
**plasmids**	**Description**	**Reference**
pE-FLP	plasmid expressing a flippase gene from the constitutive promotor pE from phage P2, AMP^R	Cui and Shearwin (2017)
pOSIP-KO	plasmid expressing 186-integrase, KAN^R	Cui and Shearwin (2017)
pOSIP-KL	plasmid expressing Lambda-integrase, KAN^R	Cui and Shearwin (2017)

Table S2: Parameters for RT-qPCR

Assay	Orientation	Sequence (5'-3')	Tm [°C]	Amplicon [bp]	compatible with *E. coli* CM strains
hsdS_short	forward	AGATACATCAAGAGCAACCGTTGTACG	61	189	CM05, CM13, CM56
	reverse	GATTGTACCTCTGGTGCTAGTTGAAACC	61		
hsdM	forward	GTTGCCAATCCACCTTATAGTGCG	61	205	CM05, CM13, CM19, CM56, CM57
	reverse	ACACCTTCAGCTGCGCCAC	63		
hsdS_long_023	forward	GCAAACTCGACCGACAAATCGAATTG	61	138	CM56, CM57
	reverse	CCCATTCTGGATAATCATTCCCATTCTCATC	61		
hsdS_long_324	forward	GCTCGGAAGAACAGGGATAATGTCACC	62	209	CM05, CM13, CM19
	reverse	GTTCTTCAAACGCAGGCATCATAATAGGC	62		
cysG	forward	CGGCGGTGGTGATGTCG	61	198	CM05, CM13, CM19, CM56, CM57
	reverse	GCGTCATCATCCGTCGCTG	63		
hcaT	forward	CGAACCAGCGCCAACCAC	61	200	CM05, CM13, CM19, CM56, CM57
	reverse	GGCTCGGTGGCGTTTGTC	63		
recA	forward	GGCCTTGCGGCACGTATG	61	200	CM05, CM13, CM19, CM56, CM57
	reverse	GCGCCGATACGACGGATG	62		

Table S3: proteomic expression of methyltransferases and restriction enzymes in two selected *S. xylosus* strains (TMW 2.1023 and TMW 2.1523). Label free quantification (LFQ) intensity values in (log_2) and mean of 3 replicates determined under planktonic growth in TSB-Lac⁺ (1% Glucose, acidified to pH 6 (lactic acid)) are indicated. The data was derived from a whole proteome analysis conducted by Schiffer et al. (42).

	Locus_Tag	annotation	LFQ intensity
TMW 2.1023	JGY91_01640	type I restriction modification subunit M	not detected
	JGY91_13160	type I restriction endonuclease subunit S	29.10
	JGY91_13165	type I restriction modification subunit M	32.20
	JGY91_13170	type I restriction endonuclease subunit S	27.65
	JGY91_13175	type I restriction endonuclease subunit R	29.51
TMW 2.1523	JGY88_00145	DEAD/DEAH box helicase (type IIG)	30.32

Table S4: base modification and motif analysis output generated by SMRT link for whole genome sequenced *S. xylosus* strains. In grey are motifs that are probably artifacts / non-genuine as the mean modification QV values are mostly below 50.

strain	motifString	type	centerPos	modificationType	fraction	nDetected	nGenome	meanQV	meanCoverage
TMW 2.1693	GCTCA	III	5	m6A	0.88	2779	3158	416.4	317.4
	GACN$_5$TGT	I	2	m6A	0.86	649	759	402.2	316.7
	ACAN$_5$GTC	I	3	m6A	0.86	656	759	391.1	314.1
TMW 2.1704	-								
TMW 2.1780	GGGTNA	II	6	m6A	0.91	2191	2410	326.4	239.7
TMW 2.1023	TCAN$_6$CTC	I	3	m6A	1.00	652	652	84.1	51.6
	GAGN$_6$TGA		2	m6A	1.00	651	652	79.3	49.4
TMW 2.1324	GCATC	II	3	m6A	0.99	4572	4595	132.7	91.6
	GATGC		2	m6A	0.99	4574	4595	128.9	91.6
	ACCN$_5$RTGT	I	1	m6A	1.00	597	597	122.3	90.5
	ACAYN$_5$GGT		3	m6A	0.98	586	597	126.0	91.6
	GATGCAVY		3		0.41	268	648	41.4	93.8
	SGGTAVYDNB		2		0.22	210	926	46.6	98.1
TMW 2.1521	GGGTNA	II	6	m6A	1.00	2354	2356	166.9	118.5
	GGGTRA		1		0.68	754	1114	62.3	122.4
	GGGTAAYD		2		0.55	142	257	49.9	122.2
	DNNNNNNGGGTAM		8		0.63	94	149	51.5	127.2
	GGGTYA		1		0.19	241	1242	45.3	129.6
	GGGTYAAAW		2		0.61	57	93	50.3	130.0
	GGGTNAAV		7		0.25	140	558	40.1	122.8
	SGTATAVCR		3		0.28	61	221	47.9	124.2
	KTTTATACY		2		0.20	58	283	43.7	132.1
	AAATATANYA		2		0.16	78	495	39.9	122.5
TMW 2.1523	GGGTNA	II	6	m6A	0.81	1866	2300	160.5	113.8
	GGGTRA		1		0.57	610	1077	62.3	118.2
TMW 2.1602	CACCG	III ?	4	(?)	0.92	1590	1728	64.5	95.2
	SGTRTAVCR		3		0.26	112	426	45.3	115.5

Table S5: RT-qPCR gene expression analysis C_q values of *hsdM*, *hsdS_short*, *hsdS_long* and reference genes *cysG*, *hcaT* and *recA* are shown.

Assay	Sample	Mean C_q Assay	Mean C_q reference genes cysG	hcaT	recA	ΔC_q *vs.* cysG	hcaT	recA	all ref genes
hsdM	**CM05**	14.79	22.89	24.48	21.91	8.10	9.69	7.12	8.31
	**CM13**	12.52	20.64	24.18	20.71	8.13	11.67	8.19	9.33
	**CM19**	12.59	19.60	23.59	20.39	7.01	11.00	7.80	8.60
	**CM56**	11.24	18.86	23.73	20.46	7.62	12.48	9.21	9.77
	**CM57**	14.63	22.39	24.45	21.91	7.77	9.83	7.29	8.29
***hsdS*_short**	**CM05**	14.41	22.89	24.48	21.91	8.49	10.07	7.50	8.69
	**CM13**	12.51	20.64	24.18	20.71	8.13	11.67	8.20	9.33
	**CM56**	11.70	18.86	23.73	20.46	7.16	12.02	8.76	9.31
***hsdS*_023**	**CM56**	13.46	18.86	23.73	20.46	5.40	10.27	7.00	7.55
	**CM57**	15.28	22.39	24.45	21.91	7.12	9.18	6.64	7.64
***hsdS*_324**	**CM05**	15.79	22.89	24.48	21.91	7.10	8.69	6.12	7.31
	**CM13**	14.02	20.64	24.18	20.71	6.62	10.16	6.69	7.82
	**CM19**	14.33	19.60	23.59	20.39	5.28	9.26	6.06	6.87

Table S6: Relative gene expression of *hsdM*, and *hsdS* between different *E. coli* constructs based on RT-qPCR gene expression analysis.

		fold change normalized to *cysG*	*hcaT*	*recA*	all
hsdM	CM19 *vs.* CM5	0.47	2.47	1.60	1.23
	CM19 *vs.* CM13	0.46	0.63	0.76	0.60
	CM57 *vs.* CM56	1.11	0.16	0.26	0.36
***hsdS*_long**	CM19 *vs.* CM5	0.28	1.49	0.96	0.74
	CM19 *vs.* CM13	0.39	0.54	0.65	0.52
	CM57 *vs.* CM56	3.28	0.47	0.78	1.06
